# Synthetic *Shewanella‐Bacillus* Microbial Consortia for Enhanced Metal Oxide Leaching From Spent Lithium‐Ion Batteries Using Brewing Wastewater as Leaching Agent

**DOI:** 10.1002/advs.77699

**Published:** 2026-09-13

**Authors:** Baocai Zhang, Chunxiao Qiao, Huichun Liu, Yiyun Wang, Yan Liu, Huan Yu, Junqi Zhang, Chao Li, Deguang Wu, Qulai Tang, Zhiwen Wang, Danni Zhang, Dake Xu, Feng Li, Hao Song

**Affiliations:** ^1^ College of Life and Health Sciences Northeastern University Shenyang China; ^2^ State Key Laboratory of Synthetic Biology School of Synthetic Biology and Biomanufacturing Tianjin University Tianjin China; ^3^ Department of Brewing Engineering Moutai Institute Renhuai Guizhou China; ^4^ Key Laboratory of Ministry of Education for Protection and Utilization of Special Biological Resources in Western China School of Life Sciences Ningxia University Yinchuan China; ^5^ School of Materials Science and Engineering Northeastern University Shenyang China

**Keywords:** *Bacillus subtilis*, brewing wastewater, extracellular electron transfer, microbial consortia, *Shewanella oneidensis*, spent LIBs recycling, synthetic biology

## Abstract

The rapid proliferation of lithium‐ion batteries (LIBs) underscores the need for recycling, yet existing methods are costly, inefficient, and environmentally challenging. This work developed a spent LIBs recycling approach using brewing wastewater and designed a microbial consortium as a biocatalyst for facilitating metal oxide leaching. The *Shewanella oneidensis* reduces spent LIBs cathode LiNi_x_Co_y_Mn_1‐x‐y_O_2_ (NCM) via extracellular electron transfer (EET) by feeding with brewing wastewater's organics, and the *Bacillus subtilis* removes residual ethanol to eliminate inhibition on EET. The catalytic performance is further enhanced via boosting EET, increasing metal oxide leaching, which, however, impairs cell viability. To bolster resistance, the global transcriptional factor IrrE is introduced, achieving a leached metal ion concentration of 102.4 mg L^−1^, 30.12 times higher than in brewing wastewater. Cellular electrophysiology analyses reveal that *S. oneidensis* utilizes *c*‐type cytochromes MtrC and OmcA, and riboflavin to transfer electrons to NCM. This study proposes a sustainable “treating waste with waste” technology for recycling spent LIBs.

## Graphical abstract

A recycling approach of spent lithium‐ion batteries is developed using brewing wastewater as the leaching agent and an engineering microbial consortium as the biocatalyst. *Shewanella oneidensis* reduces spent LiNi_x_Co_y_Mn_1‐x‐y_O_2_ cathodes via extracellular electron transfer, while *Bacillus subtilis* removes residual ethanol to eliminate inhibition. This work establishes a sustainable “treating waste with waste” strategy for metal recovery.

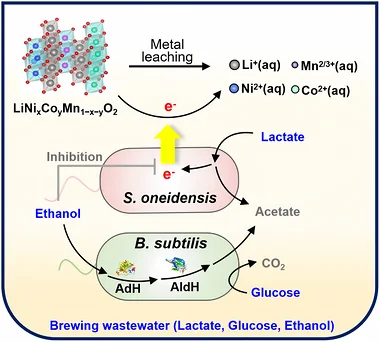

## 1 Introduction

Lithium‐ion batteries (LIBs) are used in a wide range of applications, including communications, transportation and electrical cars etc., [1], which drive unprecedented growth in LIBs production [2], resulting in over 11 million tons of spent LIBs are thus globally going to be produced by 2030 [3, 4, 5], thus posing serious environmental risks [6]. Meanwhile, the International Energy Agency (IEA) warns that LIBs supply chains need to grow tenfold by 2030, which risk depletion of over 70% of global cobalt reserves and underscores the unsustainability of LIBs [7, 8]. On the other hand, the content of these metallic elements in spent LIBs is substantially higher than that found in natural ores [9, 10, 11]. Consequently, the recycling of metals from the spent LIBs is of considerable environmental, social, and economic significance.

The methods for spent LIBs recycling include direct regeneration, pyrometallurgy, and (bio)hydrometallurgy [12, 13]. Direct regeneration of spent LIBs restores electrode materials without full decomposition and resynthesis [8, 13, 14], which avoids expensive purification but requires complex pretreatments, thus limiting scalability [4, 15]. Pyrometallurgy processes spent LIBs into metal alloys through smelting [16, 17]. Despite its operational simplicity and high metal recovery efficiency, pyrometallurgy demands extreme temperatures, produces toxic gases, and incurs high infrastructure and energy costs. (Bio)Hydrometallurgy leaches metal ions from spent LIBs cathode materials (e.g., Li‐Ni*_x_* Co*_y_* Mn_z_O_2_, NCM, and LiCoO_2_, LCO) using aqueous leaching agents, which provides many benefits, including high efficiency, better selectivity, and lower energy consumption [4, 18]. The leaching agent played a vital role in metal oxide leaching [19, 20], and a variety of leaching agents were developed, including inorganic acids (e.g., HCl [21] and H_2_SO_4_ [22]), organic acids (e.g. oxalic acid [23], citric acid [24], and malic acid [25]), as well as alkalis (e.g., NaOH [26] and NH_3_·H_2_O [27]). Meanwhile, to enhance metal oxide leaching, (bio)hydrometallurgy commonly necessitates the addition of reductants, such as hydrogen peroxide (H_2_O_2_), which is used to reduce poorly soluble high‐valence metal ions (e.g., Ni^3+^, Co^3+^ and Mn^4+^) to their more soluble lower‐valence counterparts (e.g., Ni^2+^, Co^2+^ and Mn^2/3+^) [15, 28, 29, 30]. The consumption of large amounts of reductants dramatically increases the (bio)hydrometallurgy treatment cost [19, 20], thus, it is necessary to investigate alternative methods (e.g., deep eutectic solvents [31] and contact‐electro‐catalysis [32]) to reduce the reliance on these costly reductants.

Notably, the organic matter in wastewater, which is rich in carbohydrates, organic acids, alcohols, and other compounds, contains abundant electron‐rich functional groups, such as hydroxyl, carboxyl, and aldehyde moieties [33]. These functional groups significantly impart reducing capabilities, enabling the organic matters to donate electrons to various oxidants, including metal oxides (e.g., MnO_2_ [34] and Fe_2_O_3_ [35]), metal ions (e.g., Cu^2+^ [36], and Cr^6+^ [37]), molecular oxidants (e.g., O_3_ [38]), and reactive oxygen species (e.g., ·OH [39]). The intrinsic reducing equivalents present in this organic matter make wastewater a promising and sustainable alternative to conventional reductants, which not only eliminates the need for costly reductants but also enables high‐value utilization of wastewater, thereby providing a viable approach for achieving cost‐effective and sustainable recycling of spent LIBs. To the best of our knowledge, this strategy has been largely overlooked by previous studies.

In this study, a proof‐of‐concept study on spent LIBs recycling using brewing wastewater as the leaching agent was conducted. The organic matter (e.g., lactate and glucose) in the brewing wastewater was utilized as the reductants to reduce spent LIBs cathode materials of LiNi_x_Co_y_Mn_1‐x‐y_O_2_ (NCM) for metal oxide leaching, while only a small amount of metal ions was leached out. To this end, a synthetic microbial consortium consisting of *S. oneidensis* MR‐1 and *B. subtilis* 168 was designed based on “division of labor”, in which *S. oneidensis* reduced NCM via EET using lactate, the primary organic matter in brewing wastewater, as an electron donor, and the engineered *B. subtilis* 168 removed residual ethanol in brewing wastewater via an Adh‐AldH dual enzyme cascade reaction to eliminate the ethanol inhibition on EET of *S. oneidensis*. This microbial consortium was thus used to catalyze NCM reduction to enable metal oxide leaching using the brewing wastewater, the efficiency of which could be significantly enhanced by further boosting electron shuttle riboflavin‐mediated EET of *S. oneidensis*. However, the progressive accumulation of metal oxide leaching increased cytotoxicity, decreasing cellular activity and hindering further metal oxide leaching. To bolster resistance against metal ion cytotoxicity, the global transcriptional factor IrrE was introduced into *S. oneidensis* and *B. subtilis*, resulting in total leached metal ions of 102.4 mg L^−1^, 30.12‐fold higher than those in the brewing wastewater without the microbial consortium. Finally, cellular electrophysiology analyses revealed the electron transfer mechanisms underlying the brewing wastewater‐mediated reduction of NCM catalyzed by the microbial consortium. The electrons generated from lactate catabolism in *S. oneidensis* could be directly transferred to NCM via outer membrane *c*‐Cyts MtrC and OmcA, or were first transferred to riboflavin molecules via MtrC and OmcA and then reduced NCM through riboflavin, both of which enhanced metal oxide leaching from NCM. This study demonstrated an innovative strategy for spent LIBs recycling using the brewing wastewater, enabling a sustainable “treating waste with waste” technology.

## 2 Results

### 2.1 Design of Dual‐Species Microbial Consortium Catalyzing Reduction of NCM With Brewing Wastewater for Metal Oxide Leaching

**FIGURE 1:**
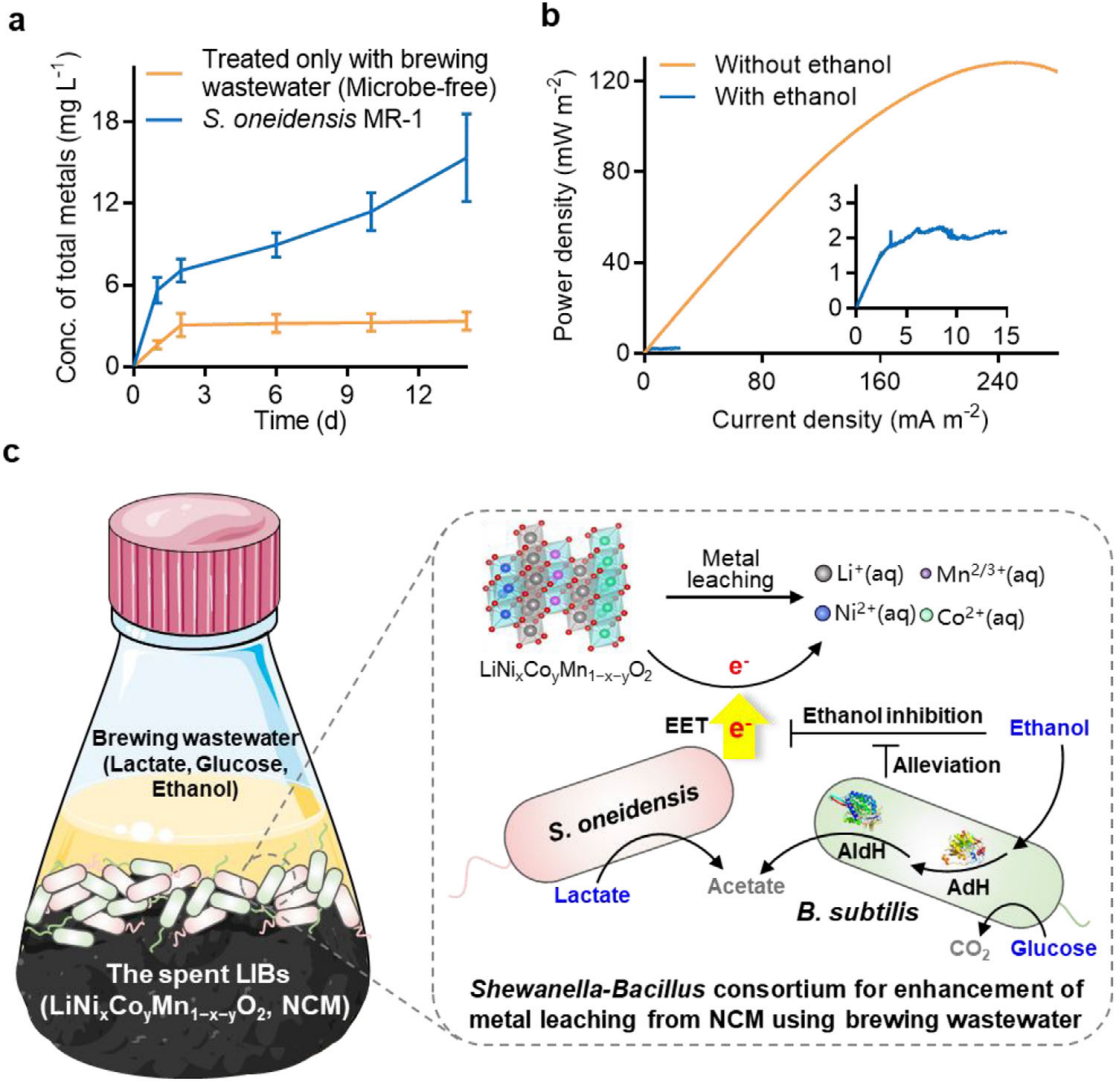
Design of a dual‐species microbial consortium consisting of *S. oneidensis* and *B. subtilis*. (a) The concentration of leached metal ions from NCM. (b) Power density curves in microbial fuel cells (MFCs) for *S. oneidensis* MR‐1 in the presence or absence of ethanol. (c) Schematic diagram of the synthetic microbial consortium consisting of recombinant *S. oneidensis* MR‐1 and *B. subtilis* 168 that catalyzes the reduction of NCM using brewing wastewater as the reductant for the purpose of metal oxide leaching.

Leaching of the metal oxide (i.e., Li, Ni, Co, and Mn) from solid‐state NCM necessitates the reduction of high‐valence metal ions (e.g., Ni^3+^, Co^3+^, Mn^4+^) to their lower‐valence counterparts (e.g., Ni^2+^, Co^2+^, Mn^2/3+^), as these ions at lower valence exhibit substantially higher solubility compared to their higher‐valence forms [4, 40]. As the dominant organic matters in brewing wastewater, lactate (−0.19 V vs. standard hydrogen electrode, SHE) and glucose (−0.05 V vs. SHE) exhibit a lower redox potential compared to Co^3+^ (1.82 V vs. SHE), Ni^3+^ (0.56–0.76 V vs. SHE), and Mn^4+^ (1.23 V vs. SHE) presented in NCM, suggesting that the reduction of NCM using brewing wastewater is thermodynamically feasible. However, when immersing the cathode material of black mass (the cathode material of spent LIBs, primarily composed of NCM, of which composition was shown in Table S1) into the brewing wastewater collected from a brewing factory in Moutai Town (Guizhou Province, China), only a very small amount of metal ions (3.4 mg L^−1^) was leached from 0.2 g L^−1^ black mass over 2 week period (Figure 1a). This was likely attributed to the dissolution of superficially bound and unstable metal oxides rather than the bulk phase reduction process. It was thus speculated that the reduction of NCM by lactate or glucose in brewing wastewater might not proceed spontaneously due to inherent reduction barriers.

To verify this hypothesis, the metal‐reducing bacterium *S. oneidensis* MR‐1 was adopted to catalyze the reduction of NCM using brewing wastewater as the reductant, in which *S. oneidensis* consumed lactate as the electron donor and reduced NCM anaerobically via extracellular electron transfer (EET). The presence of *S. oneidensis* resulted in an increase in the concentration of total leached metal ions to 15.4 mg L^−1^ (Figure 1a), a 4.53‐fold increase compared to the control group, which involved metal oxide leaching from NCM using only the brewing wastewater (3.4 mg L^−1^). These findings revealed that *S. oneidensis* overcame reaction barriers and thus initiated and enhanced the reduction of NCM by the brewing wastewater, facilitating metal oxide leaching. Despite the increased metal oxide leaching achieved by *S. oneidensis*, the leached metal content remained inadequately low, rendering this process unsuitable for applications.

Notably, the brewing wastewater typically contains residual ethanol [41]. Principal component analysis determined that the brewing wastewater used in this study contained 1.8% ethanol by volume. As ethanol is an effective antibacterial agent, it was hypothesized that the ethanol presence in the brewing wastewater could negatively impact the cell viability of *S. oneidensis*, potentially inhibiting EET. Consequently, the impact of ethanol on EET was examined. The results revealed that the EET rate, as measured by the maximum power density of a microbial fuel cell, was only 2.08 mW m^−2^ in the presence of ethanol (1.8% v/v) (Figure 1b). In contrast, in the absence of ethanol, the maximum power density of *S. oneidensis* was 128.20 mW m^−2^ (Figure 1b), which was 61.62‐fold higher than the maximum power density observed in the presence of ethanol. These findings clearly demonstrated that the residual ethanol in the brewing wastewater dramatically hindered EET of *S. oneidensis*, which would constrain its capacity to catalyze the brewing wastewater‐mediated reduction of NCM for the purpose of metal oxide leaching.

To alleviate ethanol inhibition, subsequently, a synthetic microbial consortium was developed consisting of an ethanol decomposer and a metal reducer (*S. oneidensis*) based on the principle of “division of labor”, in which the ethanol was removed by the ethanol decomposer *Bacillus subtilis*, and NCM was reduced by the metal reducer of *S. oneidensis* via the EET mechanism (Figure 1c). *B. subtilis* 168, a Gram‐positive bacterium known for its ability to withstand harsh environmental conditions such as elevated ethanol concentrations [42], was employed as the ethanol decomposer. To enhance ethanol catabolism, the alcohol dehydrogenase gene (*adh*) from *Saccharomyces cerevisiae* BY4341 and the aldehyde dehydrogenase gene (*aldH*) from *Issatchenkia terricola* XJ‐2 were co‐overexpressed under the control of the strong promoter P_43_ in *B. subtilis* 168, resulting in the recombinant strain BS1 (P_43_‐*adh*‐*aldH*, Table 1). Using this engineered *B. subtilis* strain BS1, the residual ethanol in the brewing wastewater was oxidized to acetaldehyde by alcohol dehydrogenase (Adh), which was subsequently converted into acetate catalyzed by aldehyde dehydrogenase (AldH), constituting a dual‐enzyme cascade reaction that efficiently removed ethanol, thus eliminating ethanol inhibition on the EET efficiency of *S. oneidensis*.

**TABLE 1:** Genotypes of the engineered *S. oneidensis* and *B. subtilis* strains in this study.

Strain	Genotype relevant characteristic (s)	Source
SO1	The wild‐type *Shewanella oneidensis* MR‐1 containing the plasmid pHG13, Cm^r^.	Our lab
SO2	SO1 with the P_tac_:: *ribADEHC* operon from *Bacillus subtilis* 168 integrated at pHG13, Cm^r^.	This study
SO3	SO1 with the P_tac_:: *ribADEHC* operon from *Bacillus subtilis* 168 and *irrE* gene from *Deinococcus radiodurans* R1 integrated at pHG13, Cm^r^.	This study
BS0	The wild‐type *Bacillus subtilis* 168 containing the plasmid pCE2, Cm^r^.	Our lab
BS1	BS0 with the P_43_:: *adh* gene from *Saccharomyces cerevisiae* BY4341 and *aldH* gene from *Issatchenkia terricola* XJ‐2 integrated at pCE2, Cm^r^.	This study
BSG3	The strain BSG3 was derived from recombinant *B. subtilis 168* (designated as BS120 in previous work [45]) through microbial mutation breeding and subsequent high‐yield screening aimed at further enhancing riboflavin production, the genotype of which is still unknown.	Our lab
BS2	BSG3 with the P_43_:: *adh* gene from *Saccharomyces cerevisiae* BY4341 and *aldH* gene from *Issatchenkia terricola* XJ‐2 integrated at pCE2, Cm^r^.	This study
BS3	BS2 with the P_43_:: *irrE* gene from *Deinococcus radiodurans* R1 integrated at pCE2, Cm^r^.	This study

### 2.2 Enhanced Metal Oxide Leaching by Engineering the Dual‐Species Microbial Consortia

**FIGURE 2:**
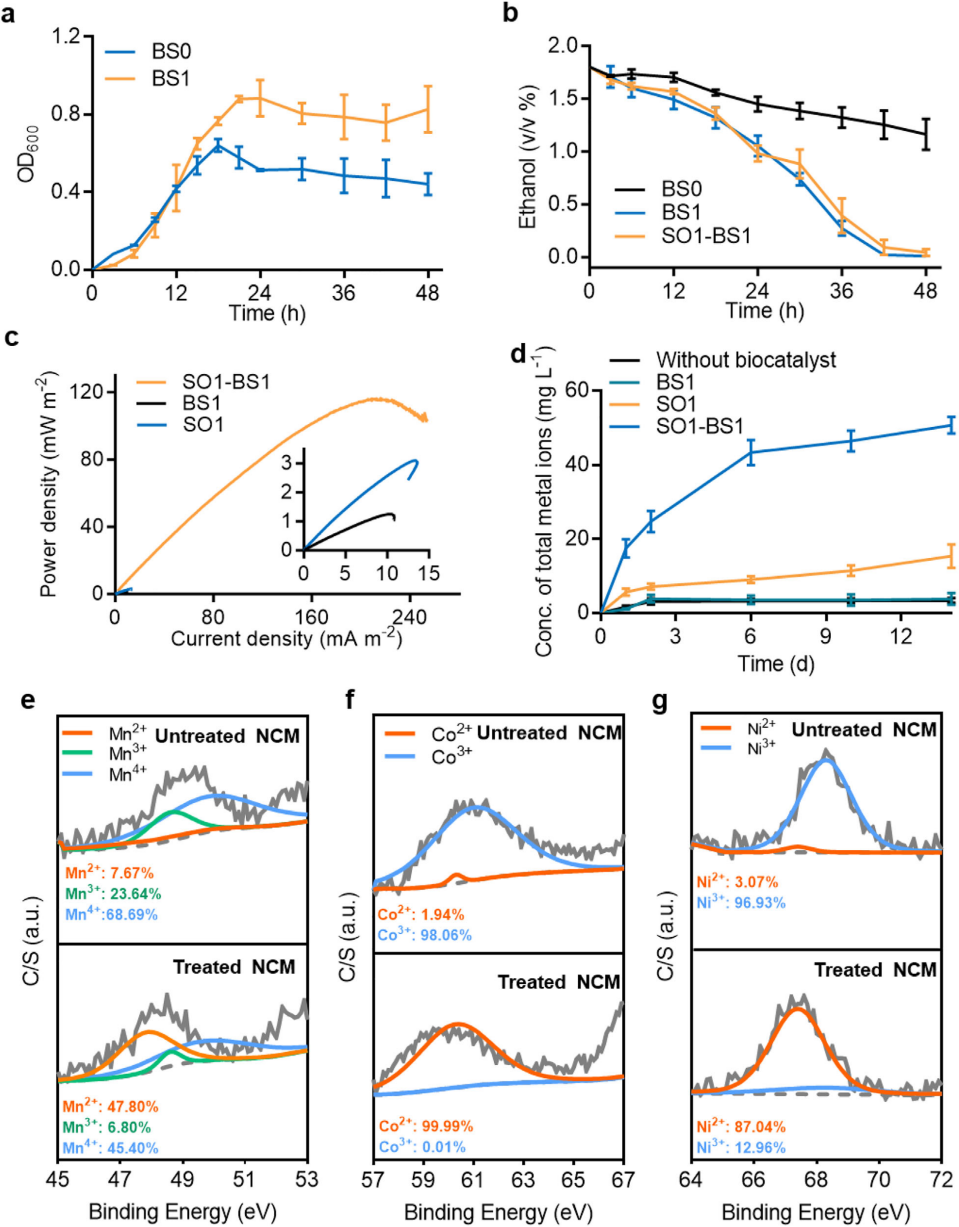
The performance of the SO1‐BS1 consortium in catalyzing the brewing wastewater‐mediated reduction of NCM to enhance metal oxide leaching. (a) Cell growth profiles of the wild‐type strain *B. subtilis* 168 (BS0) and the recombinant *B. subtilis* 168 (BS1) that overexpressed alcohol dehydrogenase Adh and aldehyde dehydrogenase AldH. (b) Volume fraction of ethanol in brewing wastewater treated with the strain BS0, strain BS1, and the SO1‐BS1 consortium. (c) Power density curves of strain SO1, strain BS1, and the SO1‐BS1 consortium. (d) Concentration of total leached metal ions from NCM catalyzed by strain SO1, strain BS1, and the SO1‐BS1 consortium. X‐ray photoelectron spectroscopy analysis of Mn 3p (e) Co 3p (f) and Ni 3p (g) in untreated NCM and brewing wastewater‐mediated reduction of NCM catalyzed by the SO1‐BS1 consortium.

The capacity of strain BS1 in ethanol removal was assessed. When cultivated with the brewing wastewater, strain BS1 achieved a higher maximum cell density (OD_600, max_ of 0.884), while the wild‐type *B. subtilis* (BS0, Table 1) exhibited OD_600, max_ of 0.641 (Figure 2a). Following 48‐h fermentation, ethanol in the brewing wastewater was effectively consumed by strain BS1, with the ethanol concentration dropping from 1.8% to 0.01% (v/v), corresponding to an ethanol removal efficiency of 99.4% (Figure 2b). In contrast, the wild‐type strain BS0 removed only approximately 35.6% of ethanol within 48 h, lowering the ethanol concentration from 1.8% to 1.16% (v/v) (Figure 2b). These findings demonstrated that the Adh‐AldH dual‐enzyme cascade reaction conferred on strain BS1 with an outstanding ethanol catabolic capacity, enabling strain BS1 to rapidly proliferate in the ethanol‐containing brewing wastewater and to efficiently remove the residual ethanol.

The microbial consortium SO1‐BS1, comprising the strains *S. oneidensis* (SO1, Table 1) and *B. subtilis* (P_43_‐*adh*‐*aldH*, BS1), was subsequently constructed. It was found that 97.6% of ethanol in the brewing wastewater could be removed by the SO1‐BS1 microbial consortium within 48 h (Figure 2b). Moreover, the maximum power density of the SO1‐BS1 consortium reached 116.15 mW m^−2^, representing 34.47‐ and 92.92‐fold increases compared to that observed in strain SO1 (3.10 mW m^−2^) and BS1 (1.25 mW m^−2^), respectively (Figure 2c), confirming that the ethanol inhibition of the EET of the SO1‐BS1 consortium was significantly alleviated.

When applying the SO1‐BS1 consortium to catalyze the brewing wastewater‐mediated reduction of NCM, 50.7 mg L^−1^ of the total leached metal ions (including Li, Ni, Co, and Mn) was leached over 2 week period (Figure 2d), in which the ionic concentrations of Li, Ni, Co, and Mn were 45.6, 2.4, 1.0, and 1.7 mg L^−1^, respectively (Figure S1), and the leaching efficiencies of Li, Ni, Co, and Mn were 57.7%, 9.5%, 11.1%, and 13.1%, respectively. In comparison to the other groups, including treating NCM using the brewing wastewater only (3.4 mg L^−1^ total leached metal ion), the brewing wastewater‐mediated reduction of NCM catalyzed by strain SO1 alone (15.4 mg L^−1^ total leached metal ion), and the brewing wastewater‐mediated reduction of NCM catalyzed by strain BS1 alone (3.8 mg L^−1^ total leached metal ion) (Figure 2d), the total leached metal ions by the SO1‐BS1 consortium increased by 14.91‐, 3.29‐, and 13.34‐fold, respectively. Thus, the microbial consortium demonstrated significantly enhanced efficiency in catalyzing the brewing wastewater‐mediated reduction of NCM for metal oxide leaching.

The oxidation states of the leached metal ions were further analyzed using X‐ray photoelectron spectroscopy (XPS). As shown in Figure 2e, deconvolution of the Mn 3p spectra in the untreated NCM revealed that Mn^4+^ (49.8 eV) and Mn^3+^ (48.6 eV) were the predominant species, accounting for 68.69% and 23.64%, respectively, with Mn^2+^ (47.8 eV) constituting only approximately 7.67%. Following treatment using brewing wastewater catalyzed by the SO1‐BS1 consortium, the levels of Mn^4+^ and Mn^3+^ decreased to 45.40% and 6.80%, whereas the level of Mn^2+^ increased to 47.80%, respectively, indicating that a substantial fraction of Mn^4+^ and Mn^3+^ in NCM was reduced. Likewise, the results of XPS also revealed the reduction of Co^3+^ and Ni^3+^ in NCM. Specifically, a significant amount of Co^3+^ (61.0 eV) was detected in the NCM, accounting for 98.06%, whereas Co^2+^ (60.3 eV) only constituted a small percentage of 1.94%. Upon treatment, almost exclusively Co^2+^ was detected based on the fitting results (Figure 2f). In terms of Ni, the percentages of Ni^2+^ (67.4 eV) and Ni^3+^ (68.3 eV) in NCM were 3.07% and 96.93%, respectively. Upon subjection to the SO1‐BS1 consortium‐enhanced reduction, Ni^3+^ accounted for only 12.96%, and Ni^2+^ accounted for 87.04% (Figure 2g). These XPS results demonstrated that the SO1‐BS1 consortium effectively catalyzed the reduction of high‐valence metal species (Mn^4^^+^, Co^3^^+^, and Ni^3^^+^) in NCM to their more soluble lower‐valence forms (Mn^2^^+^, Co^2^^+^, and Ni^2^^+^), thereby facilitating metal oxide leaching.

### 2.3 Enhancement of EET of Microbial Consortium to Facilitate Metal Oxide Leaching From NCM

**FIGURE 3:**
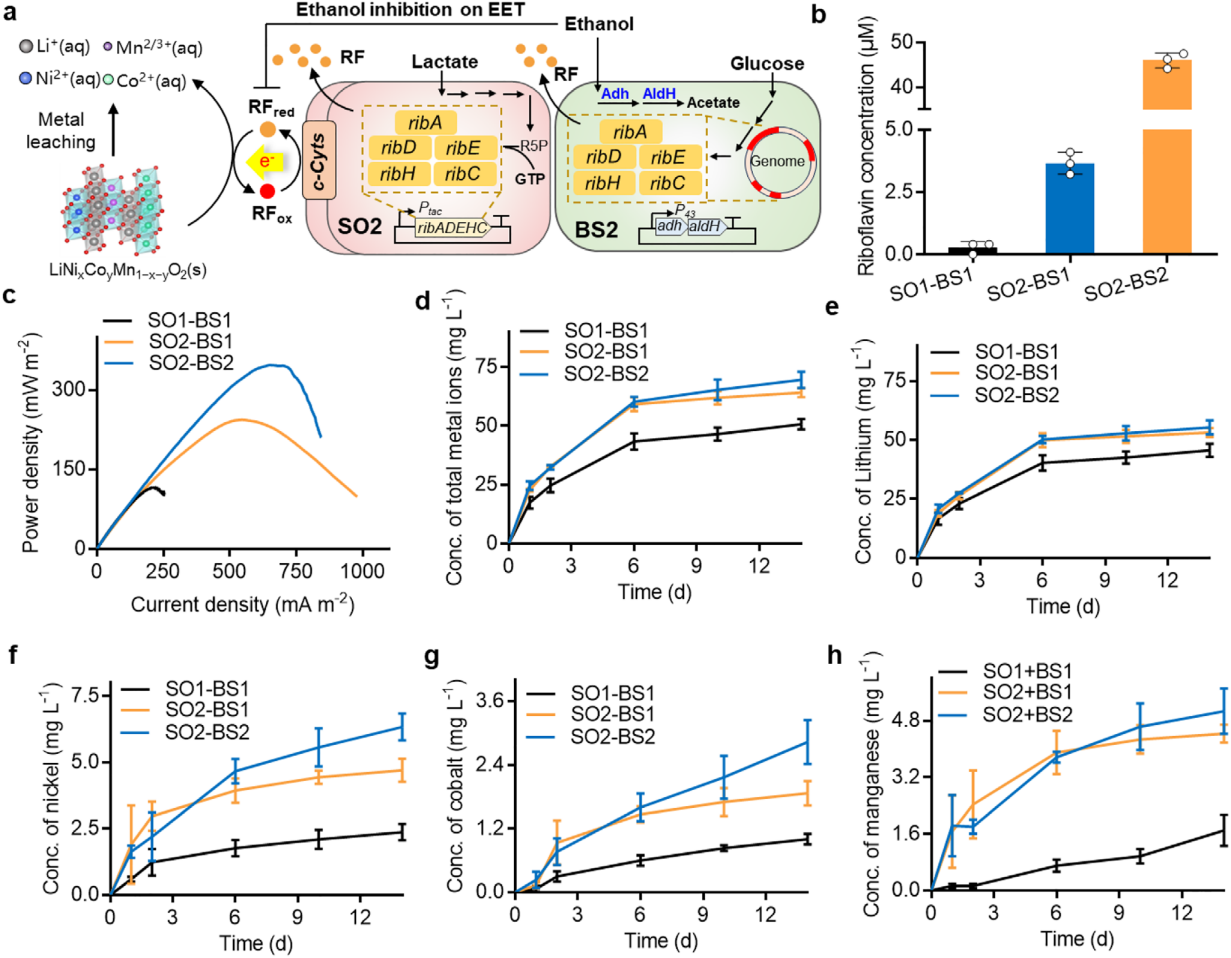
The enhancement of NCM reduction for metal oxide leaching via boosting riboflavin‐mediated EET. (a) Schematic diagram of the enhancement of riboflavin synthesis for boosting the EET of the microbial consortium. For the *S. oneidensis* MR‐1, the riboflavin synthesis gene operon was expressed in *S. oneidensis* MR‐1 (strain SO1) to construct the recombinant strain SO2. For *B. subtilis*, a recombinant *B. subtilis* 168 that was developed in our previous work was adopted, whose riboflavin synthesis was enhanced through microbial mutation breeding and screening. Abbreviations: RF, riboflavin; RF_red,_ riboflavin in reduced form; RF_ox,_ riboflavin in oxidized form; *c*‐Cyts, *c*‐type cytochromes of *S. oneidensis* MR‐1. (b) Concentration of riboflavin synthesized by the microbial consortia of SO1‐BS1, SO2‐BS1, and SO2‐BS2. (c) Power density curves of the consortia of SO1‐BS1, SO2‐BS1, and SO2‐BS2. (d) Concentration of total leached metal ions from NCM catalyzed by the consortia of SO1‐BS1, SO2‐BS1, and SO2‐BS2. The concentrations of lithium (e) nickel (f) cobalt (g) and manganese (h) leached from NCM catalyzed by the consortia of SO1‐BS1, SO2‐BS1, and SO2‐BS2.

To facilitate metal oxide leaching, the process of NCM reduction was further enhanced by improving the EET rate of *S. oneidensis* in the SO1‐BS1 consortium. *S. oneidensis* mainly employs membrane‐associated *c*‐type cytochromes (*c*‐Cyts) and the electron shuttle riboflavin to perform EET, with the EET rate responding more sensitively to changes in riboflavin concentration [43, 44]. The riboflavin biosynthesis was thus enhanced in the SO1‐BS1 microbial consortium to boost EET rate. To this end, a riboflavin biosynthesis pathway containing GTP cyclohydrolase II/ 3,4‐dihydroxy‐2‐butanone 4‐phosphate synthase (RibA), pyrimidine deaminase/reductase (RibD), riboflavin synthase (RibE), 6,7‐dimethyl‐8‐ribityllumazine synthase (RibH), and riboflavin kinase (RibC) was first overexpressed in SO1 driven by the promotor of P_tac_, resulting in the recombinant strain SO2 (P_tac_‐*ribA*‐*ribD*‐*ribE*‐*ribH*‐*ribC*) (Figure 3a and Table 1). When fed with the brewing wastewater, riboflavin synthesized by the SO2‐BS1 consortium reached 3.65 µm, 13.22‐fold higher than that synthesized by the SO1‐BS1 consortium (0.276 µm, Figure 3b). Correspondingly, the maximum power density achieved by the SO2‐BS1 consortium rose to 243.71 mW m^−2^, 2.10‐fold increase relative to the SO1‐BS1 consortium (116.15 mW m^−^^2^, Figure 3c), demonstrating an improved EET rate of the SO1‐BS1 consortium via enhanced riboflavin synthesis.

Subsequently, a recombinant *B. subtilis* strain (designated as BS120), which was developed in our previous work for efficient riboflavin synthesis [45], was adopted, and its riboflavin synthesis was further enhanced through microbial mutation breeding. A high‐yield mutant, designated as strain BSG3, was subsequently isolated via screening. Following the transfer of the plasmid pCE2‐P_43_‐*adh*‐*aldH* into BSG3, the recombinant strain BS2 (harboring genomic mutations for riboflavin synthesis and P_43_‐*adh*‐*aldH* in the pCE2 plasmid, Figure 3a and Table 1) was constructed, which was subsequently assembled with strain SO2 to form the SO2‐BS2 consortium. When fed with the brewing wastewater, the synthesized riboflavin by the SO2‐BS2 consortium reached 46.00 µM, which was 166.67‐ and 12.60‐fold higher than that of the SO1‐BS1 (0.276 µM) and SO2‐BS1 consortia (3.65 µM), respectively (Figure 3b). The maximal power density of the SO2‐BS2 consortium reached 347.29 mW m^−2^, 2.99‐ and 1.42‐fold higher than that of the SO1‐BS1 (116.15 mW m^−2^) and SO2‐BS1 consortia (243.71 mW m^−2^), respectively (Figure 3c), indicating a further improvement in the EET rate of the SO2‐BS2 consortium with increased riboflavin synthesis.

The microbial consortium was employed as a biocatalyst to catalyze the reduction of NCM using brewing wastewater for metal oxide leaching. The results showed 64.1 mg L^−1^ of the metal ions were leached when catalyzed by the SO2‐BS1 consortium, representing a 1.26‐fold increase compared to that catalyzed by the SO1‐BS1 consortium (50.7 mg L^−1^, Figure 3d). Furthermore, the maximal metal oxide leaching rate was 16.4 mg L^−1^·day^−1^ (the SO2‐BS1 consortium), 1.33 times higher than that catalyzed by SO1‐BS1 biocatalyst (12.3 mg L^−1^·day^−1^, Figure S2). Specifically, the leached concentrations of Li, Ni, Co, and Mn catalyzed by the SO2‐BS1 consortium reached 53.1, 4.7, 1.9, and 4.4 mg L^−1^, respectively, achieving leaching efficiencies of 66.5%, 18.6%, 20.2%, and 33.3% (Figure 3e–h). These results were significantly higher than those observed with the SO1‐BS1 consortium (45.6, 2.4, 1.0, and 1.7 mg L^−1^, respectively). These findings indicated that the improvement of EET in the dual‐species microbial consortium through the promotion of riboflavin synthesis enhanced its performance in catalyzing the brewing wastewater‐mediated reduction of NCM, thereby further facilitating metal oxide leaching.

In comparison, the concentration of leached metal ions achieved by the SO2‐BS2 consortium, which reached 69.6 mg L^−1^ (comprising 55.3 mg L^−1^ Li, 6.3 mg L^−1^ Ni, 2.8 mg L^−1^ Co, and 5.1 mg L^−1^ Mn), along with the maximal leaching rate of 16.2 mg L^−1^·day^−1^, was also significantly higher than those observed for the SO1‐BS1 consortium (50.7 mg L^−1^, 12.3 mg L^−1^·day^−1^) (Figure 3d–h). However, although the EET rate of the SO2‐BS2 consortium (347.29 mW m^−2^) was 43% higher than that of the SO2‐BS1 consortium (243.71 mW m^−2^) (Figure 3c), its metal oxide leaching performance (69.6 mg L^−1^, 16.2 mg L^−1^·day^−1^) did not exhibit a corresponding enhancement compared to the SO2‐BS1 consortium (64.1 mg L^−1^, 16.4 mg L^−1^·day^−1^) (Figure 3d and Figure S2). This suggested that, in the case of the SO2‐BS2 consortium, electron transfer rate might no longer constitute the primary rate‐limiting step in further accelerating metal oxide leaching.

### 2.4 Improvement of Microbial Resistance Against Metal Ion Cytotoxicity

**FIGURE 4:**
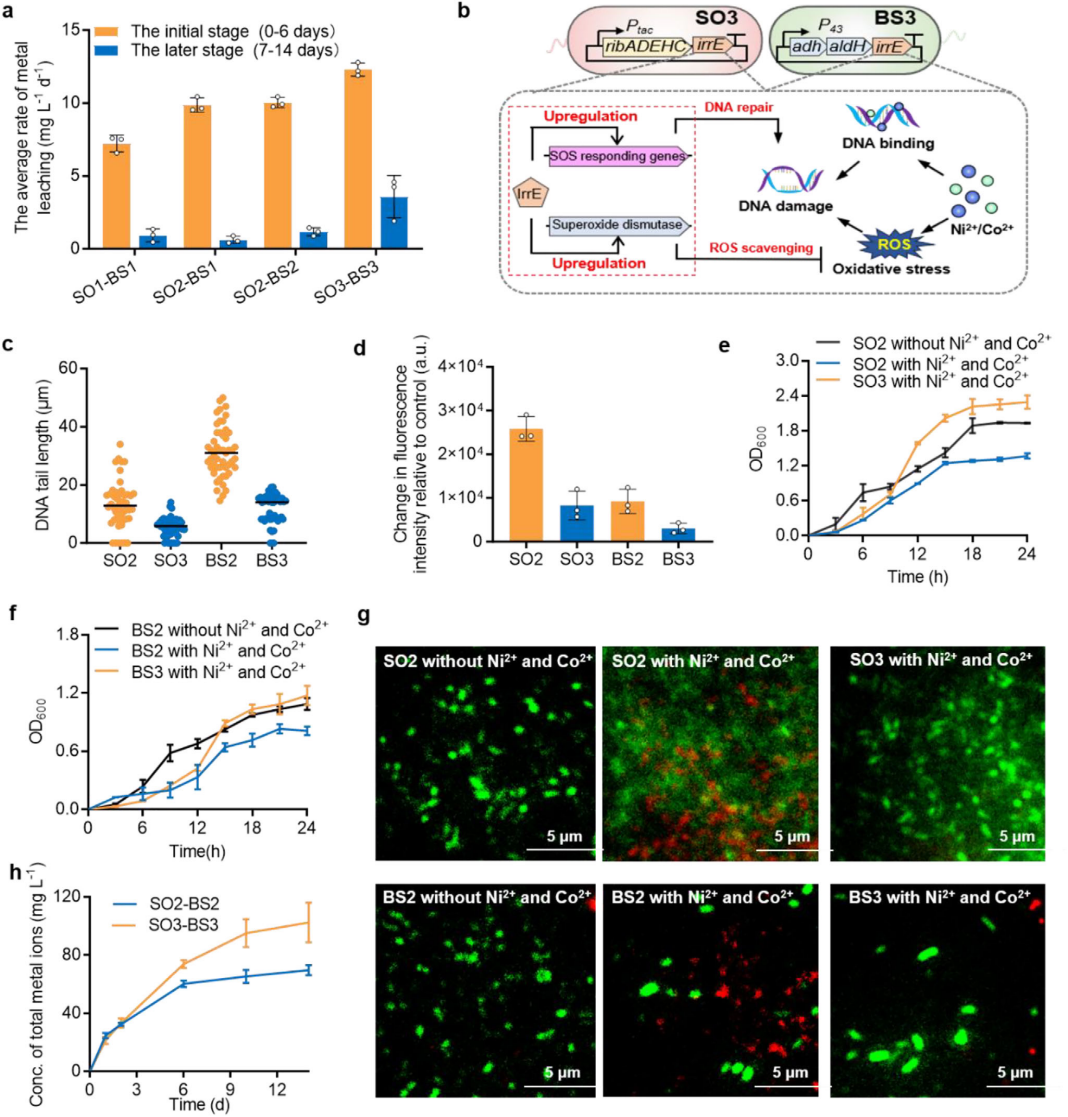
The enhancement of cell resistance against metal ion cytotoxicity via expressing the global transcriptional factor IrrE in *S. oneidensis* and *B. subtilis*. (a) The average rate of metal oxide leaching. (b) Schematic of the improvement of cell resistance against metal ion cytotoxicity via overexpressing the global transcriptional factor IrrE. Ni^2+^ and Co^2+^ can bind to adenine and guanine in DNA, catalyzing Fenton‐like reactions that produce ROS and cause DNA damage. The expression of IrrE increases superoxide dismutase levels to reduce intracellular ROS and activates the SOS response for DNA repair. (c) DNA tail length, which was measured from fluorescence images of the comet assay in Figure S3. For each experimental group, 50 cells were selected for tail length measurement. (d) Quantification of intracellular ROS levels using the CM‐H_2_DCFDA fluorescent probe. (e) Cell growth profiles of strains SO2 (in the absence of Ni^2+^ and Co^2+^), SO2 (in the presence of Ni^2+^ and Co^2+^), and SO3 (in the presence of Ni^2+^ and Co^2+^). (f) Cell growth profiles of strains BS2 (in the absence of Ni^2+^ and Co^2+^), BS2 (in the presence of Ni^2+^ and Co^2+^), and BS3 (in the presence of Ni^2+^ and Co^2+^). (g) Fluorescence microscopy images of the strains SO2 (in the absence of Ni^2+^ and Co^2+^), SO2 (in the presence of Ni^2+^ and Co^2+^), SO3 (in the presence of Ni^2+^ and Co^2+^), BS2 (in the absence of Ni^2+^ and Co^2+^), BS2 (in the presence of Ni^2+^ and Co^2+^), and BS3 (in the presence of Ni^2+^ and Co^2+^). (h) Concentration of total leached metal ions from NCM catalyzed by the SO3‐BS3 consortium. Results from three independent experiments are expressed as means and standard errors.

To further promote metal oxide leaching, attention was subsequently directed toward the cytotoxicity of leached metal ions, as prior research indicated that cell viability was highly sensitive to the metal ions released from NCM, particularly Co^2+^ and Ni^2+^ [46, 47]. An in‐depth analysis of the leaching process reflected a significant decrease in the average metal oxide leaching rate during the later stage of the reaction (days 7–14) compared with the initial 6 days for all biocatalysts (the consortia SO1‐BS1, SO2‐BS1, and SO2‐BS2). Specifically, the average leaching rates decreased from 7.222 mg L^−1^·day^−1^ to 0.921 mg L^−1^·day^−1^ for the SO1‐BS1 consortium, from 9.867 to 0.617 mg L^−1^·day^−1^ for the SO2‐BS1 biocatalyst, and from 10.033 to 1.171 mg L^−1^·day^−1^ for the SO2‐BS2 consortium, as shown in Figure 4a. It's thus speculated that, as the reaction progressed, the accumulation of leached metal ions led to increased cytotoxicity toward both *S. oneidensis* and *B. subtilis*, which impaired cell viability and catalytic activity, thereby exerting a negative feedback effect that inhibited further metal oxide leaching from NCM.

To improve the resistance of the microbial consortium against the leached metal ions, the global transcriptional factor IrrE, which originates from the extremophile *Deinococcus radiodurans* R1 [48], was overexpressed in strains SO1 and BS2, respectively, obtaining the recombinant strains SO3 (P_tac_‐*ribA*‐*ribD*‐*ribE*‐*ribH*‐*ribC*‐*irrE*) and BS3 (P_43_‐*adh*‐*aldH‐irrE*), respectively (Figure 4b and Table 1). The IrrE enables the bacterium to withstand extreme environments by repairing DNA damage [49] and scavenging intracellular reactive oxygen species (ROS) [50]. To verify these protective mechanisms, we assessed the impact of *irrE* overexpression on DNA damage and intracellular ROS levels in *S. oneidensis* cells upon exposure to 6 mg L^−1^ Ni^2+^ and 3 mg L^−1^ Co^2+^. Specifically, a comet assay was employed to evaluate DNA damage, utilizing the electrophoretic DNA tail length as a quantitative indicator of strand breakage [49]. The results revealed that *irrE* overexpression significantly relieved DNA damage, reducing DNA tail lengths by 63.9% (from 13.3 to 4.8 µm) in *S. oneidensis* and 54.2% (from 31.0 to 14.2 µm) in *B. subtilis* (Figure 4c and Figure S3). Concurrently, the CM‐H_2_DCFDA fluorescent probe analysis [51] demonstrated that *irrE* overexpression effectively scavenged intracellular ROS, decreasing fluorescence intensity by 67.8% and 67.4% in *S. oneidensis* and *B. subtilis*, respectively (Figure 4d). Collectively, these findings directly evidenced that IrrE enhanced bacterial tolerance to Ni^2+^ and Co^2+^ primarily through DNA repair and ROS scavenging.

To determine whether these molecular‐level protections correspond to enhanced cellular growth and viability, the recombinant strains were further evaluated. When exposed to 6 mg L^−1^ Ni^2+^ and 3 mg L^−1^ Co^2+^, the growth of strain SO2 was notably inhibited, with its maximum cell density decreasing from 1.941 (in the absence of Ni^2+^ and Co^2+^) to 1.367 (Figure 4e). In comparison, strain SO3, which overexpressed IrrE, attained higher cell density (OD_600, max_ = 2.291) under the identical conditions (Figure 4e). Likewise, strain BS2 experienced growth inhibition under 6 mg L^−1^ Ni^2+^ and 3 mg L^−1^ Co^2+^ stress, whereas the introduction of IrrE into strain BS3 relieved the cytotoxicity of Ni^2+^ and Co^2+^, increasing the maximum cell density of strain BS3 to 1.175 from 0.830 (strain BS2 exposed to Ni^2+^(6 mg L^−1^) and Co^2+^(3 mg L^−1^); Figure 4f). Meanwhile, Live‐dead staining also revealed that strains SO2 and BS2 exhibited more pronounced red fluorescence in the presence of Ni^2+^ and Co^2+^ (Figure 4g), indicating a higher prevalence of dead cells due to Ni^2+^ and Co^2+^ exposure. The overexpression of IrrE in strains SO3 and BS3 resulted in a noticeable reduction in the abundance of dead cells (Figure 4g), consistent with the observed growth patterns. In addition, it was found that Li^+^ (100 mg L^−1^) and Mn^2+^ (10 mg L^−1^) exhibited negligible inhibitory effects on the growth of *S. oneidensis* MR‐1 and *B. subtilis* 168, and overexpression of IrrE could not further enhance cell growth in the presence of Li^+^ or Mn^2+^ (Figure S4a,b). These results demonstrated that the leached Co^2+^ and Ni^2+^ ions exerted significant growth inhibition on the cells of *Shewanella* and *Bacillus*, and that overexpression of the *irrE* gene markedly enhanced their resistance to Co^2+^ and Ni^2+^, thus improving cell viability under Ni^2+^ and Co^2+^ stress.

The SO3‐BS3 consortium was subsequently assembled to catalyze the brewing wastewater‐mediated reduction of NCM for metal oxide leaching. In the presence of the SO3‐BS3 consortium, the total leached metal ion concentration was significantly increased. Specifically, the total leached metal ions reached 102.4 mg L^−1^, which comprised 80.8, 8.5, 5.2, and 7.9 mg L^−1^ for Li, Ni, Co, and Mn, respectively, corresponding to the leaching efficiencies of ∼100% for Li, 35.1% for Ni, 58.4% for Co, and 61.8% for Mn (Figure 4h and Figure S5). The total leached metal ions represented 1.47‐fold improvement compared to the SO2‐BS2 biocatalyst (69.6 mg L^−1^, including 55.3 mg L^−1^ Li, 6.3 mg L^−1^ Ni, 2.8 mg L^−1^ Co, and 5.1 mg L^−1^ Mn, underscoring the enhanced catalytic performance of the SO3‐BS3 consortium. Moreover, in the process of metal oxide leaching catalyzed by the SO3‐BS3 consortium, the average leaching rate in the later stage (days 7–14) increased to 3.575 mg L^−1^·day^−1^, 3.05‐fold increase compared to that of the SO2‐BS2 consortium (1.171 mg L^−1^·day^−1^) (Figure 4a). These findings demonstrated that overexpression of the *irrE* gene enhanced the resistance of *Shewanella* and *Bacillus* against the cytotoxicity of the leached Ni^2+^ and Co^2+^, which enabled the SO3‐BS3 consortium elevated and prolonged capacity of catalyzing the NCM reduction, thereby promoting the efficiency of metal oxide leaching.

### 2.5 Electron Transfer Mechanisms Underlying Brewing Wastewater‐Mediated Reduction of NCM Catalyzed by Microbial Consortia

**FIGURE 5:**
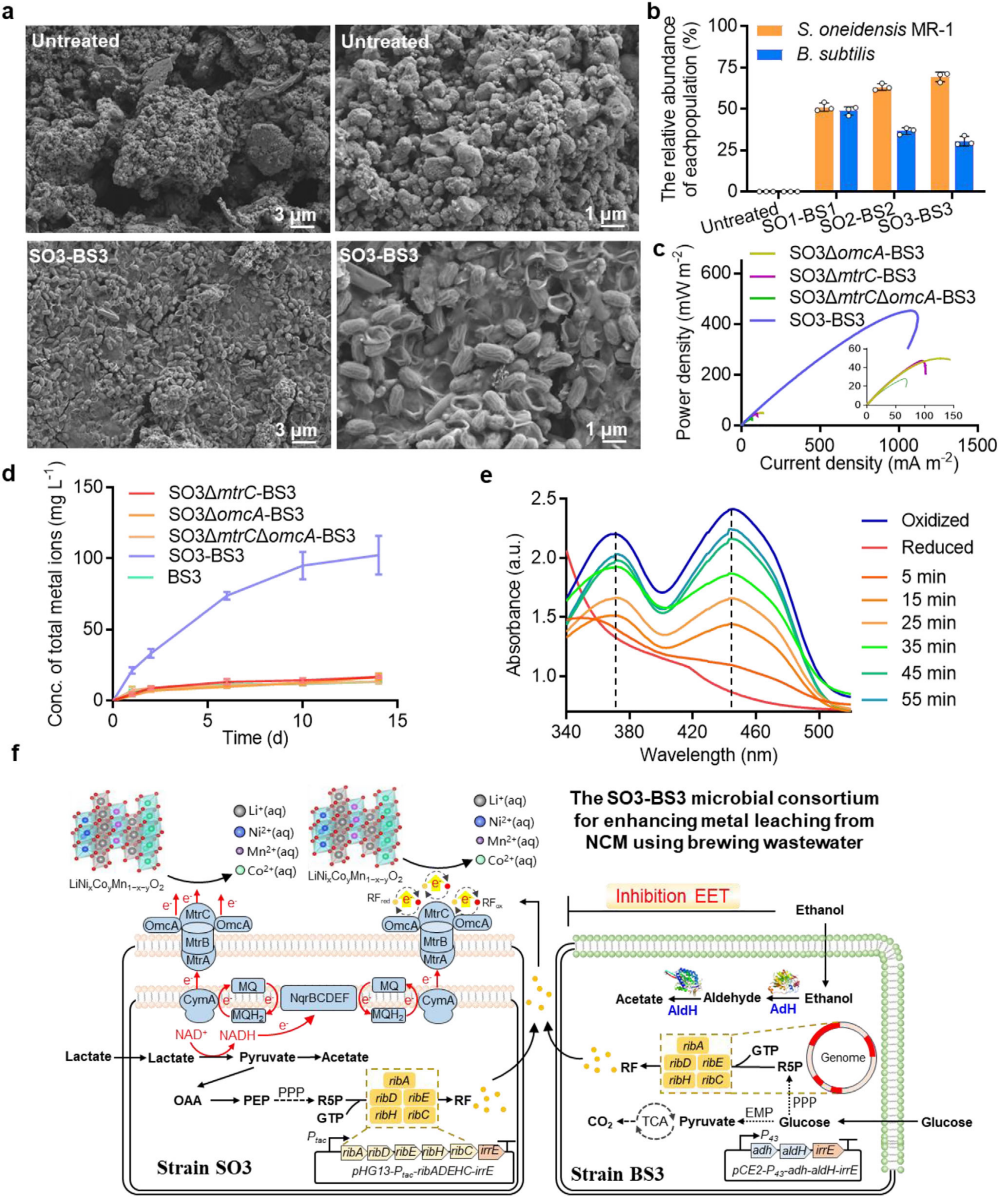
The EET mechanisms underlying brewing wastewater‐mediated reduction of NCM catalyzed by the microbial consortium. (a) SEM images of NCM. The scale bars of the SEM images were 3 and 1 µm, respectively. The reported results were checked for consistency with three individual experiments. (b) Proportion of *S. oneidensis* MR‐1 distributed on the NCM surface. (c) Power density curves of the consortia of SO3∆*mtrC*‐BS3, SO3∆*omcA*‐BS3, SO3∆*mtrC*∆*omcA*‐BS3, and SO3‐BS3. (d) Concentrations of total leached metal ions from NCM catalyzed by strain BS3, and consortia of SO3∆*mtrC*‐BS3, SO3∆*omcA*‐BS3, SO3∆*mtrC*∆*omcA*‐BS3, and SO3‐BS3. (e) The absorption spectra of riboflavin. This figure shows the absorption spectra of riboflavin in its fully oxidized and fully reduced states, as well as the spectra of reduced riboflavin after reacting with NCM anaerobically for 5, 15, 25, 35, 45, and 55 min. (f) Electron transfer mechanisms underlying brewing wastewater‐mediated reduction of NCM catalyzed by the SO3‐BS3 consortium. In the SO3‐BS3 consortium, strain BS3 converted ethanol in the brewing wastewater into acetate, removing ethanol's inhibition of the EET of strain SO3. Strain BS3 produced riboflavin from glucose, which acted as the electron shuttle to enhance EET. Meanwhile, strain SO3 catabolized lactate to generate electrons and riboflavin. The electrons produced by strain SO3 reduce NCM for metal oxide leaching, either directly via outer membrane *c*‐Cyts MtrC and OmcA or indirectly through riboflavin, thereby accelerating the leaching process. Abbreviations: AdH alcohol dehydrogenase, AldH aldehyde dehydrogenase, GTP Guanosine‐5’‐triphosphate, PPP pentose phosphate pathway, OAA oxaloacetic acid, PEP phosphoenolpyruvate, R5P ribulose‐5‐phosphate. MQ(H_2_) methyl naphthoquinone, NqrBCDEF Na^+^‐translocating NADH‐quinone reductase. MtrC and OmcA decaheme *c*‐Cyts, MtrB β‐barrel trans‐OM protein, MtrA periplasmic decaheme *c*‐Cyts, CymA inner membrane tetraheme *c*‐Cyts.

To elucidate the EET mechanism, NCM was first observed using scanning electron microscopy (SEM). It was found that a large number of cells were attached to the NCM surface when the NCM reduction was catalyzed by the SO3‐BS3 consortium (Figure 5a, SO3‐BS3), which disrupted the original NCM morphology (Figure 5a, Untreated). These cells attached to the NCM surface were isolated, and the species distribution was subsequently analyzed via colony morphology identification. This study showed that the relative abundance of *S. oneidensis* on the NCM surface was 51.14% for the SO1‐BS1 consortium, 63.28% for the SO2‐BS2 consortium, and 69.38% for the SO3‐BS3 consortium, as illustrated in Figure 5b. The higher relative abundance of *S. oneidensis* on NCM surface for SO2‐BS2 and SO3‐BS3 consortia than that of the SO1‐BS1 consortium was most likely due to the biosynthesis of riboflavin, which could facilitate *S. oneidensis* adherence to the electron acceptor (e.g., electrode in MFC) for biofilm formation [52].

Subsequently, it was explored how electrons were transferred from *S. oneidensis* to NCM for reduction. To this end, the genes encoding the critical *c*‐Cytochromes (MtrC and OmcA) in the EET conduits of *S. oneidensis* were individually or simultaneously deleted in strain SO3 using the homologous recombination method, resulting in the mutant strains SO3∆*mtrC*, SO3∆*omcA*, and SO3∆*mtrC*∆*omcA*. The results showed that the SO3∆*mtrC*‐BS3 consortium (47.67 mW m^−2^), the SO3∆*omcA*‐BS3 consortium (50.01 mW m^−2^), and the SO3∆*mtrC*∆*omcA*‐BS3 consortium (28.39 mW m^−2^) showed 9.50‐, 9.05‐, and 15.95‐fold decreases in the EET rate compared to the SO3‐BS3 consortium (452.90 mW m^−2^), respectively (Figure 5c), confirming that the disruption of MtrC and OmcA significantly inhibited the EET process in strain SO3. Correspondingly, compared with the total leached metal ions catalyzed by the SO3‐BS3 microbial consortium (102.4 mg L^−1^), the contents of leached metal ions catalyzed by the SO3∆*mtrC*‐BS3 consortium (16.7 mg L^−1^), the SO3∆*omcA*‐BS3 microbial consortium (18.7 mg L^−1^), and the SO3∆*mtrC*∆*omcA*‐BS3 consortium (13.4 mg L^−1^) were dramatically decreased by 6.13‐, 5.48‐, and 7.64‐fold, respectively (Figure 5d). Notably, the leached metal ions catalyzed by the SO3*∆mtrC*∆*omcA*‐BS3 consortium (13.4 mg L^−1^) exhibited negligible difference from that catalyzed by the sole strain BS3 (13.4 mg L^−1^), suggesting that MtrC and OmcA in *S. oneidensis* were essential for NCM reduction in metal oxide leaching.

In addition, the interaction between NCM and riboflavin was further investigated. The concentrated riboflavin underwent complete chemical reduction to its reduced form, after which the NCM suspension was introduced to conduct its anaerobic reduction. The reduction process of NCM was monitored by assessing the ratio of oxidized to reduced riboflavin, which illustrated the progressive oxidation of reduced riboflavin by NCM over time, as evidenced by the increasing absorbance peaks at 375 and 445 nm (Figure 5e), aligning with the characteristic absorption peaks of the oxidized riboflavin [53]. The intensification of these peaks as the reaction advances substantiated the ability of riboflavin to reduce NCM. Also, it was reported that MtrC and OmcA responded to the reduction of riboflavin in *S. oneidensis* [54]. Therefore, the riboflavin molecules captured electrons from the cells of *S. oneidensis* via MtrC and OmcA, which then reduced NCM and accelerated metal oxide leaching.

Based on these results, the EET mechanisms underlying the brewing wastewater‐mediated reduction of NCM catalyzed by the SO3‐BS3 consortium were refined (Figure 5f). When catalyzing the brewing wastewater‐mediated reduction of NCM for metal oxide leaching, strain BS3 in the SO3‐BS3 consortium eliminated the ethanol inhibition on EET of *S. oneidensis* by converting residual ethanol in the brewing wastewater into acetate via the Adh‐AldH dual enzyme cascade reaction. Also, strain BS3 synthesized riboflavin, which was employed as an electron shuttle for promoting the EET of *S. oneidensis*. Meanwhile, strain SO3 in the SO3‐BS3 consortium catabolized lactate for intracellular electron generation and riboflavin synthesis. The generated electrons were then used to reduce NCM via the EET mechanism. Specifically, the electrons generated from strain SO3 could be either directly transferred to NCM via outer membrane *c*‐Cyts MtrC and OmcA, or first transferred to riboflavin molecules via MtrC and OmcA. then reduced NCM by the reduced‐state riboflavin, both of which enhance metal oxide leaching from NCM.

### 2.6 Economic Viability of the Metal Oxide Leaching From Spent LIBs Using Brewing Wastewater Catalyzed by the SO3‐BS3 Consortia

**FIGURE 6:**
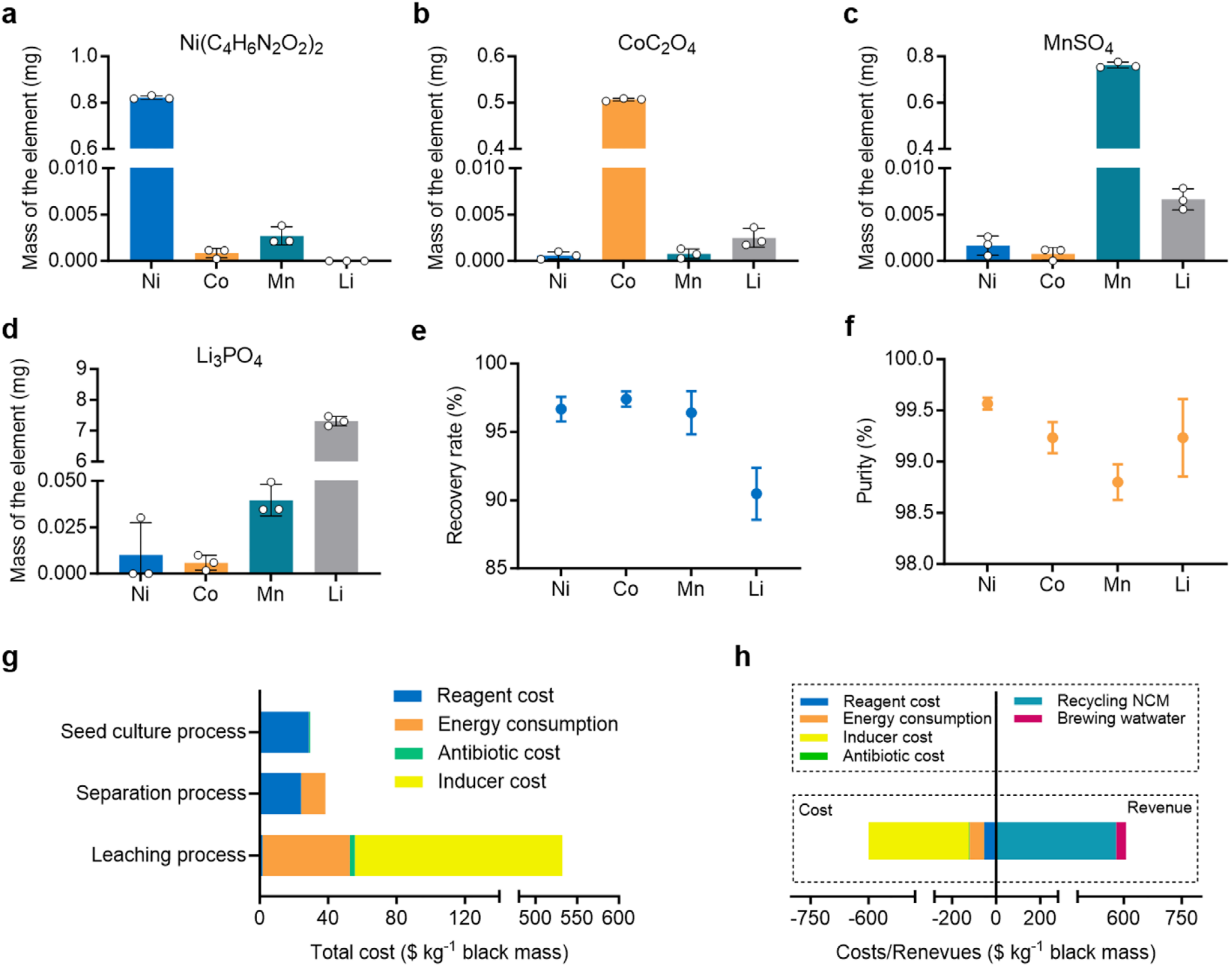
Stepwise Precipitation approach for separation and recovery of metal salts from the leachate and the economic analysis. (a) Mass of Ni, Co, Mn and Li in Ni(C_4_H_6_N_2_O_2_)_2_. (b) Mass of Ni, Co, Mn, and Li in CoC_2_O_4_. (c) Mass of Ni, Co, Mn, and Li in MnSO_4_. (d) Mass of Ni, Co, Mn, and Li in Li_3_PO_4_. (e) Recovery efficiency of the Ni, Co, Mn, and Li from the leaching solution via the Stepwise Precipitation approach. (f) Corresponding purities of the isolated Ni (Ni(C_4_H_6_N_2_O_2_)_2_), Co (CoC_2_O_4_), Mn (MnSO_4_), and Li (Li_3_PO_4_) products. (g) Detailed cost breakdown of the recovery process by process area. The costs were categorized into four types: reagent cost, energy consumption, antibiotic cost, and inducer cost. Data were calculated per kg of black mass processed. (h) Cost and revenue streams per kg of black mass processed. Negative and positive values represent costs and revenues, respectively.

To recover multiple metal ions (Li, Ni, Co, and Mn) from the leachate, a Stepwise Precipitation approach was developed for the sequential separation and purification of high‑purity metal salts, the schematic diagram of which was illustrated in Figure S6. Briefly, the separation process involved the sequential precipitation of Ni and Co using dimethylglyoxime and oxalic acid, followed by the solvent extraction of Mn utilizing saponified D2EHPA, and concluded with the final precipitation of Li using H_3_PO_4_. Through the Stepwise Precipitation approach, 0.822 mg of Ni (as Ni(C_4_H_6_N_2_O_2_)_2_, Figure 6a), 0.506 mg of Co (as CoC_2_O_4_, Figure 6b), 0.762 mg of Mn (as MnSO_4_, Figure 6c), and 7.310 mg of Li (as Li_3_PO_4_, Figure 6d) were selectively recovered from 100 mL leachate. The final recovery efficiency and purity of each metallic component were individually evaluated. The results showed that 96.7% of Ni, 97.4% of Co, 96.4% of Mn, and 90.5% of Li were recovered (Figure 6e), with the purity of 99.6%, 99.2%, 98.8%, and 99.2%, respectively (Figure 6f). These results demonstrated that this Stepwise Precipitation approach validated the feasibility of recovering high‐purity metal salts directly from complex biological leachates.

The economic feasibility of the entire process was subsequently evaluated at an industrial scale, using 1 kg of black mass of NCM as the functional unit for cost‐benefit accounting analysis. The techno‐economic parameters utilized for this assessment were provided in Table S2. Based on these fundamental parameters, the economic assessment was systematically structured around comprehensive cost inputs and revenues. On the cost side, the total operational expenses were categorized into three sequential stages, namely the seed culture, leaching, and separation processes, which comprehensively covered basal reagents (e.g., culture media components, the NCM black mass, and chemical precipitants/extractants), reagents for plasmid maintenance and gene expression (e.g., chloramphenicol and IPTG), and process‐specific energy consumption (cultivation, heating, and filtration). On the revenue side, the gross revenue was derived from two distinct streams, comprising the commercial value of the recovered high‐purity metal salts (Li_3_PO_4_, Ni(C_4_H_6_N_2_O_2_)_2_, CoC_2_O_4_, and MnSO_4_) and the environmental revenue from treating brewing wastewater.

According to the assessment (Table S2 and Note S1), the proposed technology achieved a net profit of 5.754$ kg^−1^ of NCM black mass, thus demonstrating the economic feasibility of the proposed technology. This profit margin was excellent and highly competitive in comparison to traditional chemical leaching technologies, such as HCl (∼0.2$ kg^−1^) [55] and acetic acid (∼1.105$ kg^−1^) [56]. Our system maintained strong profitability despite the costs associated with the antibiotic and inducer. The utilization of brewing wastewater contributed to this economic viability. On the one hand, it generated supplementary environmental treatment revenue. In addition, it also served as a zero‐cost complex nutrient source, completely eliminating the need for conventional culture media. Regarding the cost for engineered strain cultivation, the antibiotic (chloramphenicol in this work) accounted for only 2.4% and 0.55% of the combined costs of the seed culture and leaching processes, respectively, indicating that stable plasmid maintenance was not a major economic burden for this process (Figure 6g). Conversely, the reliance on IPTG posed a significant economic bottleneck. Our analysis revealed that IPTG accounted for an overwhelming 79.8% of the total operational costs (Figure 6h). To fully realize its industrial potential, future efforts must focus on optimizing the engineered consortia by replacing IPTG‐inducible systems with auto‐inducible or constitutive promoters, thereby completely eliminating the inducer‐associated costs.

## 3 Discussions

**FIGURE 7:**
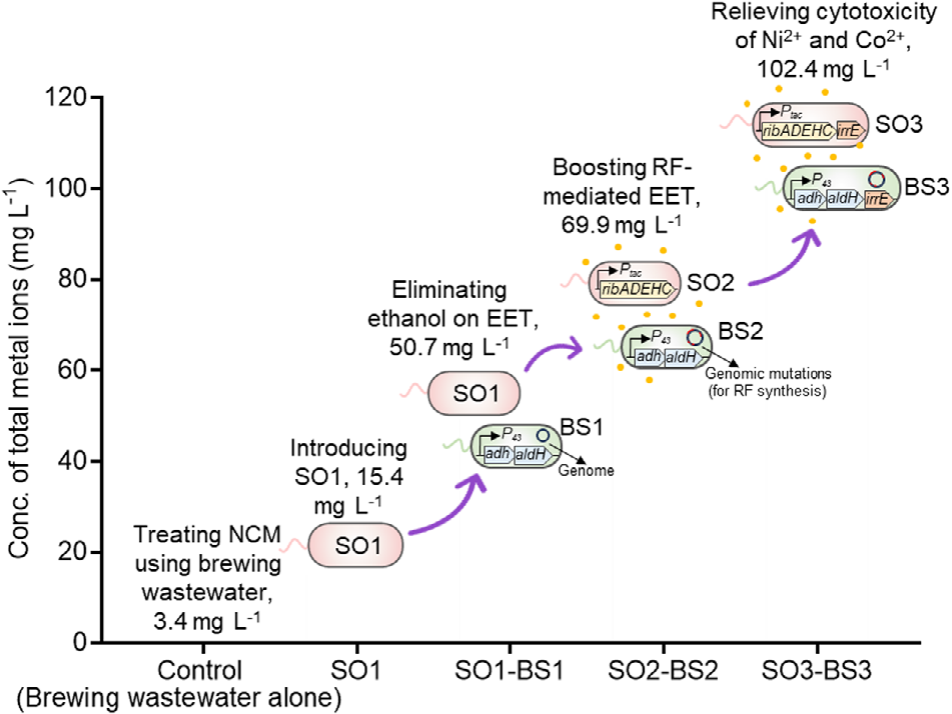
Enhancement of metal oxide leaching upon gradual optimization of the engineered *S. oneidensis* MR‐1‐*B. subtilis* 168 microbial consortia.

In this study, synthetic microbial consortia were developed based on the principle of “division of labor” for spent LIBs recycling using brewing wastewater, in which *S. oneidensis* reduced NCM via EET using the organics in the brewing wastewater as the reductants, and *B. subtilis* removed residual ethanol in brewing wastewater to eliminate ethanol inhibition on EET of *S. oneidensis*. To verify the feasibility, the brewing wastewater collected from a brewing factory in Moutai Town, Guizhou Province, China, was utilized to treat NCM anaerobically, but only a very small amount of metal ions (3.4 mg L^−1^) was leached out (Figure 7). The metal‐reducing bacterium *S. oneidensis* was subsequently introduced, resulting in a 4.53‐fold increase in leached metal ions (15.4 mg L^−1^, Figure 7), despite the fact that the EET of *S. oneidensis* was significantly inhibited by residual ethanol in brewing wastewater (Figure 1b). These findings reflected the workability of brewing wastewater‐mediated reduction of NCM for metal oxide leaching catalyzed by a metal‐reducing bacterium.

To enhance the metal oxide leaching, the alcohol dehydrogenase (Adh) and aldehyde dehydrogenase (AldH) were overexpressed (strain BS1) to remove residual ethanol via the Adh‐AldH dual enzyme cascade reaction, thereby eliminating the ethanol inhibition on the EET of strain SO1 in reducing NCM (recombinant *S. oneidensis*). The removal of ethanol significantly improved the EET rate of the SO1‐BS1 consortium (Figure 2c), leading to the total leached metal ions concentration increase from 15.4 mg L^−1^ (strain SO1 alone) to 50.7 mg L^−1^ (Figure 7). Subsequently, the EET rate of the SO1‐BS1 consortium was further improved by promoting the synthesis of the electron shuttle riboflavin (Figure 3c), resulting in total leached metal ions of 69.6 mg L^−1^ (SO2‐BS2 microbial consortium, Figure 7). However, the progressive accumulation of leached Ni^2+^ and Co^2+^ increased cytotoxicity (Figure 4b,c), which decreased cellular activity and, in turn, hindered further metal oxide leaching.

To relieve the cytotoxicity of Ni^2+^ and Co^2+^, the global transcriptional regulator IrrE was overexpressed in strains SO2 and BS2, resulting in the formation of the SO3‐BS3 consortium. The Ni^2+^ and Co^2+^ can bind to adenine and guanine bases in DNA and catalyze Fenton‐like reactions within cells, leading to the generation of ROS, thus causing DNA damage [57]. IrrE enables cells to upregulate the expression of superoxide dismutase, which is instrumental in scavenging intracellular ROS [50], and activates the cellular SOS response to facilitate DNA repair [48]. Consequently, the presence of IrrE significantly enhanced the resistance of strains SO3 and BS3 to Ni^2+^‐ and Co^2+^‐induced cytotoxicity (Figure 4b,c). This enhancement led to a substantial increase in the concentration of total leached metal ions, reaching 102.4 mg L^−1^ when catalyzed by the SO3‐BS3 consortium, which was 30.12‐fold higher than that observed in brewing wastewater without the consortium (Figure 7). Thus, these efforts achieved a substantial increase in leached metal ions from NCM. Previous study suggested that leached Ni^2+^ and Co^2+^ compromise cell viability [47, 58]. However, engineering strategies to enhance cellular resistance to Ni^2+^‐ and Co^2+^ ‐induced cytotoxicity in the context of spent LIBs recycling have not been developed. To the best of our knowledge, this study is the first to demonstrate that the global transcriptional regulator IrrE can protect cells from Ni^2+^‐induced and Co^2+^‐induced cytotoxicity, thereby significantly enhancing the leaching of metal oxides.

Comprehensive analyses of cellular electrophysiology were conducted to elucidate the EET mechanisms underlying the brewing wastewater‐mediated reduction of NCM catalyzed by microbial consortia. It was found that the outer membrane *c*‐Cyts MtrC and OmcA in *S. oneidensis* were essential for the reduction of NCM for metal oxide leaching (Figure 5d). Additionally, riboflavin in its reduced form could be oxidized by NCM (Figure 5e). Therefore, the electron transfer mechanisms were refined (Figure 5f). In the process of the brewing wastewater‐mediated reduction of NCM catalyzed by the SO3‐BS3 consortium, strain BS3 eliminated the ethanol inhibition on the EET of *S. oneidensis* by removing residual ethanol and synthesized riboflavin as the electron shuttle. Strain SO3 catabolized lactate for intracellular electron generation and riboflavin biosynthesis. The generated electrons were then used to reduce NCM for metal oxide leaching via EET. Specifically, the electrons generated from strain SO3 could be either directly transferred to NCM via outer membrane *c*‐Cyts MtrC and OmcA, or first transferred to riboflavin via MtrC and OmcA, then used to reduce NCM by the reduced‐state riboflavin. These two EET mechanisms underlie the enhanced metal oxide leaching from NCM.

We systematically benchmarked our approach against both traditional chemical leaching (inorganic/organic acids) and conventional bioleaching (bacterial and fungal). Briefly, inorganic acids (e.g., H_2_SO_4_ and HCl) and organic acids (e.g., citric acid, malic acid, and acetic acid), when used in combination with exogenous reducing agents, typically achieved leaching efficiencies exceeding 90% for all target metals [25, 59, 60, 61, 62] (Table S3). In bioleaching systems, bacterial processes employing *Acidithiobacillus thiooxidans* or *Acidithiobacillus ferrooxidans* generally yielded Li recoveries in the range of 31%–82%, while Ni, Co, and Mn efficiencies remained below 37% [63, 64, 65] (Table S4). Fungal‐based bioleaching using *Aspergillus niger* achieved markedly higher performance, with Li extraction reaching 93%–100%, Mn 70%–90%, and Ni and Co 38%–87% under optimized conditions [66, 67] (Table S4). Our proposed microbial consortium achieved leaching efficiencies of ∼100% for Li, 35.1% for Ni, 58.4% for Co, and 61.8% for Mn. While the recovery of Li was highly competitive, the leaching efficiencies for the transition metals (Ni, Co, and Mn) remained lower than those achieved by inorganic acids, organic acids, and fungal bioleaching systems.

Despite lacking a prominent edge in transition metal extraction at this proof‐of‐concept stage, our “treating waste with waste” strategy presented substantial operational, economic, and environmental advantages over these established technologies. Conventional chemical leaching (inorganic or organic) typically required elevated temperatures (50°C–80°C) [59, 68], posed risks of corrosive gas emissions (e.g., Cl_2_ [60] or SO_x_ [69]), and inherently relied on high‐concentration acids alongside costly chemical reducing agents like H_2_O_2_ [70]. Similarly, traditional bioleaching systems demanded energy‐intensive aeration, continuous mechanical agitation, and expensive addition of exogenous culture media. In contrast, our synthetic consortium operated anaerobically under mild ambient conditions (30°C) without the addition of any chemical reductants. By directly utilizing brewing wastewater as a zero‐cost nutrient source and leaching agent, the leaching process in our work completely eliminated the energy burden of aeration, the cost of supplementary reductants, and the generation of strongly acidic secondary effluents. This perfectly aligned with circular economy principles and demonstrated highly robust economic feasibility. Ultimately, our process achieved an excellent net profit of 5.754$ kg^−1^ of NCM black mass, which was substantially higher than the profit margins typically obtained from traditional acid leaching methods.

It should be recognized that while the present work established a robust proof‐of‐concept at the laboratory scale, several lines of investigation were still required to translate this approach into a practically viable industrial process. First, the leaching efficiencies for transition metals (Ni, Co, and Mn) through the proposed approach in this work remained lower than those of chemical processes. Therefore, key operational parameters, such as the inoculation ratio of *S. oneidensis* to *B. subtilis*, the pulp density of the NCM black mass, and cultivation strategies (e.g., fed‐batch or continuous operations), needed to be systematically optimized to maximize metal oxide leaching kinetics and overall efficiencies. Second, to eliminate the costs associated with antibiotics and inducers during large‐scale applications, the expression cassettes of the functional genes should be integrated directly into the host chromosomes under the control of constitutive promoters. This genomic integration will completely obviate the need for antibiotic addition for plasmid maintenance and IPTG supplementation for gene expression, which not only significantly reduces operational costs but also guarantees biosafety and regulatory compliance. In addition, the long‐term stability and reproducibility of the microbial consortium at a larger scale needed to be thoroughly considered, in particular under the condition of natural fluctuations in raw brewing wastewater compositions (e.g., varying organic loads or pH levels across different batches).

## 4 Conclusions

This study developed microbial consortia comprising engineered *S. oneidensis* and *B. subtilis*, which enabled efficient recycling of spent LIBs utilizing brewing wastewater as the leaching agent to achieve metal oxide leaching. Through synthetic biology engineering strategies, the EET rate of the microbial consortia was promoted by enhancing biosynthesis of riboflavin, thereby further enhancing the leaching of metal oxides. Nevertheless, the progressive accumulation of leached Ni^2+^ and Co^2+^ adversely affected cell viability, consequently impeding further metal oxide leaching. To address this challenge, the global transcriptional factor IrrE was overexpressed to enhance resistance to the cytotoxicity of Ni^2+^ and Co^2+^, resulting in a significant increase in the concentration of leached metal ions, reaching 102.4 mg L^−1^, which was 30.12‐fold higher than that achieved with brewing wastewater alone. Besides, the EET mechanisms underlying the brewing wastewater‐mediated reduction of NCM catalyzed by microbial consortia were elucidated, revealing that electrons generated from *S. oneidensis* could be transferred to NCM either via MtrC and OmcA directly, or via the electron shuttle riboflavin indirectly. This study highlighted the potential for recycling spent LIBs using organic matter‐bearing wastewater, and thus achieving a sustainable “treating waste with waste” technology.

## 5 Materials and Methods

### 5.1 Gene Synthesis and Plasmid Construction

The genes for alcohol dehydrogenase, aldehyde dehydrogenase, and a global transcriptional factor were screened and extracted from the NCBI database. After gene sequence codon optimization with an online tool (http://www.jcat.de/), the designed open reading frame with promoter and RBS was synthesized in vitro and assembled into plasmids pCE2 or pHG13, respectively. Plasmid pCE2 and its derivatives were transformed into *Bacillus subtilis* 168 strain (ATCC33234). Construction of plasmid pHG13 and its derivatives was performed in *E. coli* WM3064. The *E. coli* WM3064 was employed to transfer plasmids into *Shewanella oneidensis* MR‐1 strain (ATCC700550) through conjugation. All strains used in this experiment were from strains kept in our laboratory.

### 5.2 Culture of the *B. subtilis‐S. oneidensis* Consortium and Dissolution of LIBs

To establish a consortium of *B. subtilis* and *S. oneidensis*, both microorganisms were initially cultured in Luria‐Bertani (LB) medium containing 10 g L^−1^ NaCl, 5 g L^−1^ yeast extract, and 10 g L^−1^ peptone (Aladdin, Shanghai), which was supplemented with 0.2 g L^−1^ chloramphenicol for plasmid. Overnight cultures of each strain were subsequently inoculated into a medium formulated with brewing water collected from a brewing factory in Moutai Town (Guizhou) at a 1% (v/v) ratio, induced by the isopropyl‐β‐d‐thiogalactoside (IPTG, 0.5 mm, Heowns, Tianjin) for gene expression. Following a brief period of subculturing, the culture was transferred into anaerobic bottles, supplemented with 0.2 g L^−1^ of black mass (Tianzhongbao, Beijing), and purged with air for 15 min. Anaerobic fermentation was then conducted at 30°C for a period of 14 days to facilitate the dissolution of LIBs. Upon completion of the dissolution process, samples were collected to quantify the concentrations of dissolved lithium (Li), nickel (Ni), cobalt (Co), and manganese (Mn) from the LIBs in the liquid medium. Sampling occurred at various time intervals (0, 1, 2, 6, 10, and 14 days). To isolate the dissolved metals, centrifugation was performed at 13 500 rpm for 1 min. The resulting solutions were subsequently diluted with 2% HNO_3_ and 10% H_2_O_2_ to remove organic matter, and the pH was adjusted to 3–4 using the NaOH solution in preparation for inductively coupled plasma optical emission spectrometry (ICP‐OES) analysis. Each experiment was conducted in triplicate.

### 5.3 Microbial Fuel Cell Setup

An H‐type two‐chamber cell with a working volume of 140 mL was employed to establish a microbial fuel cell, wherein a Nafion 117 membrane served as the proton exchange membrane, effectively separating the anode and cathode compartments. In the anodic chamber, a carbon cloth measuring 1 cm × 1 cm functioned as the anodic electrode. The bacterial cells cultured in brewing wastewater were introduced into the anode compartment. The cathodic electrode consisted of a carbon cloth measuring 2.5 cm × 3 cm, and a solution of 50 mm potassium ferrocyanide in 50 mm phosphate buffer (composed of 50 mm K_2_HPO_4_ and 50 mm KH_2_PO_4_, Aladdin, Shanghai) was utilized as the cathodic electron donor. The output voltages across a 2 kΩ resistor were recorded at a temperature of 30°C.

### 5.4 Electrochemical Analyses

Using a CHI1000C multichannel electrochemical workstation (CH Instrument, Shanghai, China), the linear sweep voltammetry (LSV) analysis was employed to obtain the polarization curves for maximum power density and maximum current density calculation. In LSV, the scan rate was set to 0.1 mV s^−1^. The maximum power density (P) and current density (I) were calculated using Equations S1 and S2, respectively.

(1) $$P=\frac{V\times I}{S}\;$$

(2) $$I=\frac{I_{P}}{S}\;$$

where V is the voltage; I_p_ is the maximum current in the polarization curve, and S is the projected area of the anode.

### 5.5 Analysis of the Inhibitory Effect of Ethanol on EET

*S. oneidensis* MR‐1 was initially cultured in LB medium. Overnight culture was added to a test medium at a 1% (v/v) ratio, used to set up the microbial fuel cell and electrochemical analyses as described in Materials and Methods 5.3–5.4. For the analysis of the inhibitory effect of ethanol on EET, the test medium contained 15 g L^−1^ lactate, 4 g L^−1^ glucose, 6.78 g L^−1^ Na_2_HPO_4_, 3 g L^−1^ KH_2_PO_4_, 0.5 g L^−1^ NaCl, 1 g L^−1^ NH_4_Cl, 0.1 mm MgSO_4_, 0.1 mm CaCl_2_ (Aladdin, Shanghai) and 1.8% (v/v) ethanol (Controlled group was without ethanol).

### 5.6 Ethanol Measurement

The ethanol degradation by bacteria was analyzed using the Biological Sensing Analyzer (SBA‐40D, Biology Institute of Shandong Academy of Sciences, China). An overnight bacterial culture was inoculated at 1% (v/v) into 100 mL of brewing wastewater and incubated at 30°C with shaking at 250 rpm. Samples were taken every 6 h over a 48‐h period. Each sample was centrifuged at 10 000 rpm to collect the supernatant, which was diluted with deionized water to 1/5 and subsequently filtered through a 0.22 µm aqueous‐phase filter membrane. Before testing, the analyzer was calibrated with deionized water and 1% ethanol.

### 5.7 Riboflavin Measurement

Riboflavin concentration was determined using an HPLC system (Shimadzu, Japan). A 1 mL aliquot of the bacterial culture was centrifuged to retain the supernatant. Subsequently, 200 µL of the supernatant was filtered through a 0.22 µm aqueous filter membrane to prepare the HPLC sample. The analysis of riboflavin was conducted using an Aminex HPX‐87H column (300 mm × 7.8 mm), performed using a Shim‐pack GIS C18 column (250 mm × 2.5 mm, 5 µm) with a mobile phase of 30% methanol and 70% 0.1% NaH_2_PO_4_. A UV detector at 260 nm was used, with the column at 35°C, a flow rate of 1 mL min^−1^, and an injection volume of 10 µL.

### 5.8 Analyses of the Surface of Black Mass Before and After the Reaction

For solid‐state analysis, residual solids were extracted from the reactor via centrifugation. The samples underwent two sequential washings with distilled water and ethanol. Subsequently, the washed samples were subjected to vacuum freeze‐drying. The oxidation states of both pristine and reacted black mass of NCM were characterized using X‐ray photoelectron spectroscopy (XPS, ESCALAB XI+, Thermo Scientific, America). Particular attention was given to the analysis of the 3p orbitals of Ni, Co, and Mn, as these elements demonstrate significant variation in their oxidation states. Energy calibration of the XPS data was conducted using the C 1s peak. Morphological characteristics were examined using a field‐emission scanning electron microscope (FE‐SEM, JSM‐7900F, Jeol).

### 5.9 Cell Viability Quantification

A single bacterial colony was inoculated into 5 mL of LB medium and incubated overnight. Subsequently, the overnight culture was introduced at a 1% (v/v) inoculum ratio into the test medium as described in Materials and Methods 5.5 without ethanol. This medium was supplemented with either 6 mg L^−1^ of Ni^2+^ and 3 mg L^−1^ of Co^2+^, or 100 mg L^−1^ of Li^+^ and 10 mg L^−1^ of Mn^2+^. Samples were taken over a 48‐h period. The optical density of the bacterial suspension at 600 nm was determined using a UV‐visible spectrophotometer (T6, Persee, Beijing). Following the determination of the growth curve, cells were centrifuged (5000 rpm, 4°C, 10 min) and collected, then washed and resuspended in 0.9% NaCl. They were stained for 15 min with the LIVE/DEAD *Bac*Light Bacterial Viability Kits (Thermo Scientific, America) at room temperature, protected from light. Using a fluorescence microscope (Leica Inverted Fluorescence Microscope DMi1), the stained cells were observed: SYTO 9 dye emits green light at 500 nm under 480 nm excitation, and PI dye emits red light at 635 nm under 490 nm excitation.

### 5.10 NCM Reduction of Riboflavin

Complete reduction of riboflavin was achieved using a 100 mL H‐type two‐chamber microbial fuel cell. The anodic chamber contained a riboflavin solution with a platinum net electrode, while the cathodic chamber contained a PB buffer, a platinum net electrode, and an Ag/AgCl reference electrode. After purging with N_2_ for 30 min, riboflavin was reduced at −0.80 V under stirring, as confirmed by spectrophotometry. NCM reduction experiments were conducted under anoxic conditions at 25°C using riboflavin solutions. The experiments utilized 100 mL glass vials, which were shielded from light to prevent photochemical side reactions. The experimental solution was prepared by diluting NCM stock suspensions into the riboflavin solution to achieve a final NCM concentration of 0.2 g L^−1^, followed by a 1‐h reaction period under continuous stirring. Throughout the experiments, 0.9‐mL aliquots of the suspension were extracted from the reactors using plastic syringes. The reaction in these aliquots was promptly quenched by removing NCM particles through 0.22 µm syringe filtration, and the absorbance was rapidly measured within the 340 to 540 nm range.

### 5.11 Determination of Population of Bacteria

The anaerobic bottles were incubated at 4°C for one hour. Subsequently, the culture medium was carefully decanted, and sterile saline was introduced into the anaerobic bottle. The mixture was vortexed to dissociate the cells. A serial dilution of the bacterial suspension was performed, and it was subsequently plated onto solid culture media to facilitate single colony enumeration.

### 5.12 Homologous Recombination and Construction of Mutant Strains

Deletions of the genes *mtrC* and *omcA* in *S. oneidensis* were achieved via a two‐step homologous recombination strategy utilizing the suicide plasmid pHG1.0. To construct the recombinant deletion plasmids, the ∼500 bp upstream and ∼500 bp downstream flanking fragments of each target gene were amplified via PCR using *S. oneidensis* genomic DNA as the template. These upstream and downstream fragments were subsequently fused via overlap extension PCR and cloned into the restriction sites of the plasmid pHG1.0. The resulting constructs were transformed into *E. coli* WM3064, a diaminopimelic acid (DAP)‐auxotrophic donor strain cultured in LB medium supplemented with 0.3 mm DAP. Biparental conjugation was conducted by mixing the *E. coli* donor and *S. oneidensis* recipient cells on DAP‐supplemented LB agar. For the first‐step selection, single‐crossover transconjugants were isolated on nutrient agar containing 30 µg mL^−1^ gentamicin but lacking DAP to eliminate the donor cells. To initiate the second‐step crossover event, verified single‐crossover colonies were cultured in antibiotic‐free LB broth and subsequently plated onto LB agar supplemented with 10% (w/v) sucrose for *sacB*‐mediated counter‐selection. Sucrose‐resistant and gentamycin‐sensitive colonies were screened, and the successful *mtrC*‐ and *omcA*‐deleted mutants were verified via colony PCR and subsequent Sanger sequencing. The deletion of the genes *mtrC* and *omcA* was performed in the wild‐type strain *S. oneidensis* MR‐1, which was subsequently transformed with the plasmid pHG13‐P_tac_‐*ribADEHC*‐*irrE*, resulting in the mutant strains SO3∆*mtrC*, SO3∆*omcA*, and SO3∆*mtrC*∆*omcA*.

### 5.13 Comet Assay for DNA Damage

Bacterial cells in the mid‐logarithmic growth phase were subjected to exposure to 6 mg L^−1^ of Ni^2+^ and 3 mg L^−1^ of Co^2+^ for durations of 8 and 6 h for *S. oneidensis* and *B. subtilis*, respectively. 10 µL of the cell suspension was combined with 100 µL of a 0.5% low‐melting agarose (LMA) solution. Subsequently, 50 µL of this mixture was dispensed onto a Comet assay microscope slide (Beyotime, Shanghai) and evenly distributed within a well. Following solidification at 4°C, a layer of 0.5% lysozyme‐LMA was applied atop the gel and allowed to solidify. The prepared slide was then incubated for 30 min at 30°C for *S. oneidensis* and 37°C for *B. subtilis*. The slide underwent sequential immersion in a lysing solution (composed of 2.5 M NaCl, 100.0 mm EDTA, 10.0 mm Tris‐HCl, 1% sodium N‐lauryl sarcosine, 0.6% Triton X‐100, pH 10.0) for 1 h in darkness, followed by immersion in an enzyme digestion solution (comprised of 2.5 m NaCl, 10.0 mm EDTA, 10.0 mm Tris‐HCl, and 0.5 mg mL^−1^ proteinase K, pH 7.4) at 37°C for 2 h. Electrophoresis was conducted in an electrophoresis buffer (sodium acetate and Tris at pH 9.0) at 12 V for 30 min under chilled conditions. Subsequently, the slide was allowed to dry at room temperature in darkness and was stained with 50.0 mL of 1.0 mm YOYO‐1 in 5% DMSO. After air‐drying in the dark for 5 min, the stack of microgel was imaged with a fluorescence microscope (Ex/Em 491/509 nm). ImageJ was used to analyze the DNA tail lengths.

### 5.14 Fluorescence Assays on Intracellular ROS

Intracellular ROS levels were measured using CM‐H_2_DCFDA (Beyotime, Shanghai). Bacterial cells were harvested by centrifugation (8000 rpm, 5 min), washed twice with sterile PBS, and resuspended to an optical density at OD_600_ of 0.5. The cells were then incubated with CM‐H_2_DCFDA at a final concentration of 10 µm in the dark at 30°C (*S. oneidensis*) or 37°C (*B. subtilis*) for 30 min. After probe loading, the cells were washed with sterile PBS to remove extracellular probe and resuspended in fresh PBS. For ROS induction, cells were treated with 6 mg L^−1^ of Ni^2+^ and 3 mg L^−1^ of Co^2+^ for 5 h. Untreated cells served as the negative control. Following treatment, the cells were transferred to a black 96‐well plate, and the fluorescence intensity was measured using a microplate reader (Ex/Em 495/530 nm). Fluorescence readings were normalized to the OD_600_ (cell density) of each sample. All measurements were performed in triplicate, and the relative ROS level was expressed as the change relative to the untreated control.

### 5.15 The Stepwise Precipitation Approach for Separation and Recovery of Metal Salts

0.2 mol L^−1^ dimethylglyoxime solution was first added to the leachate at a stoichiometric molar ratio of 2:1 (dimethylglyoxime: Ni) to selectively precipitate Ni as Ni(C_4_H_6_N_2_O_2_)_2_. From the resulting filtrate, Co was subsequently precipitated as CoC_2_O_4_, by introducing 0.5 mol L^−1^ oxalic acid solution at the molar ratio of 1.2:1 (oxalic acid: Co). Both isolated precipitates were filtered. Subsequently, Mn was extracted from the solution using 70% saponified D2EHPA with 20 mmol L^−1^ HCl at an aqueous‐to‐organic phase ratio of 0.5. The Mn‐loaded D2EHPA was stripped with 0.1 mol L^−1^ H_2_SO_4_ to obtain MnSO_4_. Finally, Li was recovered from the remaining aqueous phase as Li_3_PO_4_, by adding 0.5 mol L^−1^ H_3_PO_4_ solution at the molar ratio of 1.1:1 (H_3_PO_4_: Li). To determine the elemental composition, the purified metal salts were re‐dissolved in 4 m HCl and subsequently quantified by ICP‐OES. The recovery efficiency of each metal was determined by comparing the mass of the target metal in the recovered salts with that initially present in the leachate. The purity of each recovered metal salt was calculated as the mass ratio of the target metal to the total mass of the four major metals (Li, Ni, Co, and Mn) present in the product. Other elements, such as Cu and Fe, were not considered, as their concentrations were below the detection limit.

## Author Contributions

**B.‐C.Z. and C.‐X.Q**. designed the project, analysed data, and drafted the manuscript. **B.‐C.Z., C.‐X.Q., H.‐C.L., Y.‐Y.W., Y.L, H.Y., J.‐Q.Z., C.L., D.‐G.W., Q.‐L.T., Z.‐W.W., D.‐N.Z., D.‐K.X., and F.L**. performed experiments. **H.S**. supervised the project, designed experiments, analyzed data, and critically revised the manuscript.

## Conflicts of Interest

The authors declare no conflicts of interest.

## Supporting information




**Supporting File**: advs77699‐sup‐0001‐SuppMat.docx.

## Data Availability

The data that support the findings of this study are available in the supporting information of this article.

## References

[1] J. Choi and D. Aurbach , “Promise and Reality of Post‐Lithium‐Ion Batteries With High Energy Densities,” Nature Reviews Materials 1, no. 4 (2016): 16013, 10.1038/natrevmats.2016.13.

[2] C. Bauer , S. Burkhardt , N. Dasgupta , et al., “Charging Sustainable Batteries,” Nature Sustainability 5, no. 3 (2022): 176–178, 10.1038/s41893-022-00864-1.

[3] J. Wang , J. Ma , Z. Zhuang , et al., “Toward Direct Regeneration of Spent Lithium‐Ion Batteries: A Next‐Generation Recycling Method,” Chemical Reviews 124, no. 5 (2024): 2839–2887, 10.1021/acs.chemrev.3c00884. 38427022

[4] B. Biswal , B. Zhang , P. Tran , J. Zhang , and R. Balasubramanian , “Recycling of Spent Lithium‐Ion Batteries for a Sustainable Future: Recent Advancements,” Chemical Society Reviews 53, no. 11 (2024): 5552–5592, 10.1039/d3cs00898c. 38644694

[5] H. Lv , H. Huang , C. Huang , Q. Gao , Z. Yang , and W. Zhang , “Electric Field Driven De‐Lithiation: A Strategy Towards Comprehensive and Efficient Recycling of Electrode Materials From Spent Lithium Ion Batteries,” Applied Catalysis B: Environmental 283 (2021): 119634, 10.1016/j.apcatb.2020.119634.

[6] D. Kang , M. Chen , and O. Ogunseitan , “Potential Environmental and Human Health Impacts of Rechargeable Lithium Batteries in Electronic Waste,” Environmental Science & Technology 47, no. 10 (2013): 5495–5503, 10.1021/es400614y. 23638841 PMC5920515

[7] D. Larcher and J. Tarascon , “Towards Greener and More Sustainable Batteries for Electrical Energy Storage,” Nature Chemistry 7, no. 1 (2015): 19–29, 10.1038/nchem.2085. 25515886

[8] J. Wang , K. Jia , J. Ma , et al., “Sustainable Upcycling of Spent LiCoO_2_ to an Ultra‐Stable Battery Cathode at High Voltage,” Nature Sustainability 6, no. 7 (2023): 797–805, 10.1038/s41893-023-01094-9.

[9] G. Harper , R. Sommerville , E. Kendrick , et al., “Recycling Lithium‐Ion Batteries From Electric Vehicles,” Nature 575, no. 7781 (2019): 75–86, 10.1038/s41586-019-1682-5. 31695206

[10] T. Yang , D. Luo , X. Zhang , et al., “Sustainable Regeneration of Spent Cathodes for Lithium‐Ion and Post‐Lithium‐Ion Batteries,” Nature Sustainability 7, no. 6 (2024): 776–785, 10.1038/s41893-024-01351-5.

[11] E. Fan , L. Li , Z. Wang , et al., “Sustainable Recycling Technology for Li‐Ion Batteries and Beyond: Challenges and Future Prospects,” Chemical Reviews 120, no. 14 (2020): 7020–7063, 10.1021/acs.chemrev.9b00535. 31990183

[12] X. Ma , Z. Meng , M. Bellonia , et al., “The Evolution of Lithium‐Ion Battery Recycling,” Nature Reviews Clean Technology 1, no. 1 (2025): 75–94, 10.1038/s44359-024-00010-4.

[13] P. Li , S. Luo , Y. Lin , et al., “Fundamentals of the Recycling of Spent Lithium‐Ion Batteries,” Chemical Society Reviews 53, no. 24 (2024): 11967–12013, 10.1039/d4cs00362d. 39471089

[14] Z. Ju , T. Zheng , B. Zhang , et al., “Magnetically Oriented Nanosheet Interlayer for Dynamic Regeneration in Lithium Metal Batteries,” Proceedings of the National Academy of Sciences 121, no. 44 (2024): 2413739121, 10.1073/pnas.2413739121. PMC11536145 39441637

[15] J. Neumann , M. Petranikova , M. Meeus , et al., “Recycling of Lithium‐Ion Batteries—Current State of the Art, Circular Economy, and Next Generation Recycling,” Advanced Energy Materials 12, no. 17 (2022): 2102917, 10.1002/aenm.202102917.

[16] M. Zhou , B. Li , J. Li , and Z. Xu , “Pyrometallurgical Technology in the Recycling of a Spent Lithium Ion Battery: Evolution and the Challenge,” ACS ES&T Engineering 1 (2021): 1369, 10.1021/acsestengg.1c00067.

[17] A. Holzer , L. Wiszniewski , S. Kern , and H. Raupenstrauch , “Optimization of a Pyrometallurgical Process to Efficiently Recover Valuable Metals From Commercially Used Lithium‐Ion Battery Cathode Materials LCO, NCA, NMC622, and LFP,” Metals 12 (2022): 1642, 10.3390/met12101642.

[18] Y. Li , W. Lv , H. Huang , et al., “Recycling of Spent Lithium‐Ion Batteries in View of Green Chemistry,” Green Chemistry 23, no. 17 (2021): 6139–6171, 10.1039/d1gc01639c.

[19] V. Srivastava , V. Rantala , P. Mehdipour , et al., “A Comprehensive Review of the Reclamation of Resources From Spent Lithium‐Ion Batteries,” Chemical Engineering Journal 474 (2023): 145822, 10.1016/j.cej.2023.145822.

[20] J. Roy , S. Rarotra , V. Krikstolaityte , et al., “Green Recycling Methods to Treat Lithium‐Ion Batteries E‐Waste: A Circular Approach to Sustainability,” Advanced Materials 34, no. 25 (2022): 2103346, 10.1002/adma.202103346. 34632652

[21] M. Joulie , R. Laucournet , and E. Billy , “Hydrometallurgical Process for the Recovery of High Value Metals From Spent Lithium Nickel Cobalt Aluminum Oxide Based Lithium‐Ion Batteries,” Journal of Power Sources 247 (2014): 551–555, 10.1016/j.jpowsour.2013.08.128.

[22] K. Chan , J. Anawati , M. Malik , and G. Azimi , “Closed‐Loop Recycling of Lithium, Cobalt, Nickel, and Manganese From Waste Lithium‐Ion Batteries of Electric Vehicles,” ACS Sustainable Chemistry & Engineering 9, no. 12 (2021): 4398–4410, 10.1021/acssuschemeng.0c06869.

[23] X. Zeng , J. Li , and B. Shen , “Novel Approach to Recover Cobalt and Lithium From Spent Lithium‐Ion Battery Using Oxalic Acid,” Journal of Hazardous Materials 295 (2015): 112–118, 10.1016/j.jhazmat.2015.02.064. 25897692

[24] J. Choi , J. Kim , S. Kim , and Y. Yun , “Simple, Green Organic Acid‐Based Hydrometallurgy for Waste‐To‐Energy Storage Devices: Recovery of NiMnCoC_2_O_4_ as an Electrode Material for Pseudocapacitor From Spent LiNiMnCoO_2_ Batteries,” Journal of Hazardous Materials 424 (2022): 127481. 34666292 10.1016/j.jhazmat.2021.127481

[25] E. Fan , J. Yang , Y. Huang , et al., “Leaching Mechanisms of Recycling Valuable Metals From Spent Lithium‐Ion Batteries by a Malonic Acid‐Based Leaching System,” ACS Applied Energy Materials 3, no. 9 (2020): 8532–8542, 10.1021/acsaem.0c01166.

[26] W. Wang , Y. Han , T. Zhang , L. Zhang , and S. Xu , “Alkali Metal Salt Catalyzed Carbothermic Reduction for Sustainable Recovery of LiCoO_2_: Accurately Controlled Reduction and Efficient Water Leaching,” ACS Sustainable Chemistry & Engineering 7, no. 19 (2019): 16729–16737, 10.1021/acssuschemeng.9b04175.

[27] J. Yu , B. Ma , Z. Qiu , C. Wang , and Y. Chen , “Separation and Recovery of Valuable Metals From Ammonia Leaching Solution of Spent Lithium‐Ion Batteries,” ACS Sustainable Chemistry & Engineering 11, no. 26 (2023): 9738–9750, 10.1021/acssuschemeng.3c01714.

[28] J. Cong , S. Luo , Q. Sun , et al., “Study on Deep Synergistic Leaching Mechanism of Spent Lithium Iron Phosphate Under the H_2_SO_4_‐H_2_O_2_ System and Precise Chemical Precipitation Recovery Strategy of Lithium Carbonate,” Separation and Purification Technology 363 (2025): 132326, 10.1016/j.seppur.2025.132326.

[29] W. Wang , Y. Zhang , X. Liu , and S. Xu , “A Simplified Process for Recovery of Li and Co From Spent LiCoO_2_ Cathode Using Al Foil as the In Situ Reductant,” ACS Sustainable Chem Eng 7, no. 14 (2019): 12222, 10.1021/acssuschemeng.9b01564.

[30] M. Chen , X. Ma , B. Chen , et al., “Recycling End‐of‐Life Electric Vehicle Lithium‐Ion Batteries,” Joule 3, no. 11 (2019): 2622–2646, 10.1016/j.joule.2019.09.014.

[31] M. Tran , M. Rodrigues , K. Kato , G. Babu , and P. Ajayan , “Deep Eutectic Solvents for Cathode Recycling of Li‐Ion Batteries,” Nature Energy 4, no. 4 (2019): 339–345, 10.1038/s41560-019-0368-4.

[32] H. Li , A. Berbille , X. Zhao , Z. Wang , W. Tang , and Z. Wang , “A Contact‐Electro‐Catalytic Cathode Recycling Method for Spent Lithium‐Ion Batteries,” Nature Energy 8, no. 10 (2023): 1137–1144, 10.1038/s41560-023-01348-y.

[33] B. Logan and K. Rabaey , “Conversion of Wastes Into Bioelectricity and Chemicals by Using Microbial Electrochemical Technologies,” Science 337, no. 6095 (2012): 686–690, 10.1126/science.1217412. 22879507

[34] B. Tebo , J. Bargar , B. Clement , et al., “BIOGENIC MANGANESE OXIDES: Properties and Mechanisms of Formation,” Annual Review of Earth and Planetary Sciences 32, no. 1 (2004): 287–328, 10.1146/annurev.earth.32.101802.120213.

[35] M. Ahmed and L. Lin , “Ferric Reduction in Organic Matter Oxidation and its Applicability for Anaerobic Wastewater Treatment: A Review and Future Aspects,” Reviews in Environmental Science and Bio/Technology 16, no. 2 (2017): 273–287, 10.1007/S11157-017-9424-3.

[36] A. Pham , A. Rose , and T. Waite , “Kinetics of Cu (II) Reduction by Natural Organic Matter,” The Journal of Physical Chemistry A 116, no. 25 (2012): 6590, 10.1021/jp300995h. 22574891

[37] J. Cao , Z. Cui , T. Wang , et al., “Reductive Removal of Cr (VI) by Citric Acid Promoted by Ceramsite Particles: Kinetics, Influential Factors, and Mechanisms,” Materials Today Communications 28 (2021): 102716, 10.1016/j.mtcomm.2021.102716.

[38] X. Jin , S. Peldszus , and P. Huck , “Reaction Kinetics of Selected Micropollutants in Ozonation and Advanced Oxidation Processes,” Water Research 46, no. 19 (2012): 6519–6530, 10.1016/j.watres.2012.09.026. 23079129

[39] M. Cheng , G. Zeng , D. Huang , et al., “Hydroxyl Radicals Based Advanced Oxidation Processes (AOPs) for Remediation of Soils Contaminated With Organic Compounds: A Review,” Chemical Engineering Journal 284 (2016): 582–598, 10.1016/j.cej.2015.09.001.

[40] W. Lv , Z. Wang , H. Cao , Y. Sun , Y. Zhang , and Z. Sun , “A Critical Review and Analysis on the Recycling of Spent Lithium‐Ion Batteries,” ACS Sustainable Chemistry & Engineering 6, no. 2 (2018): 1504–1521, 10.1021/acssuschemeng.7b03811.

[41] L. Braeken , B. Bruggen , and C. Vandecasteele , “Regeneration of Brewery Waste Water Using Nanofiltration,” Water Research 38, no. 13 (2004): 3075–3082, 10.1016/j.watres.2004.03.028. 15261546

[42] S. Romero , E. Merino , F. Bolivar , G. Gosset , and A. Martinez , “Metabolic Engineering of Bacillus Subtilis for Ethanol Production: Lactate Dehydrogenase Plays a Key Role in Fermentative Metabolism,” Applied and Environmental Microbiology 73, no. 16 (2007): 5190, 10.1128/AEM.00625-07. 17586670 PMC1950962

[43] Y. Yang , Y. Ding , Y. Hu , et al., “Enhancing Bidirectional Electron Transfer of Shewanella oneidensis by a Synthetic Flavin Pathway,” ACS Synthetic Biology 4, no. 7 (2015): 815–823, 10.1021/sb500331x. 25621739

[44] E. Marsili , D. Baron , I. Shikhare , D. Coursolle , J. Gralnick , and D. Bond , “Shewanella Secretes Flavins That Mediate Extracellular Electron Transfer,” Proceedings of the National Academy of Sciences 105, no. 10 (2008): 3968–3973, 10.1073/pnas.0710525105. PMC2268775 18316736

[45] G. Wang , T. Shi , T. Chen , et al., “Integrated Whole‐Genome and Transcriptome Sequence Analysis Reveals the Genetic Characteristics of a Riboflavin‐Overproducing Bacillus Subtilis,” Metabolic Engineering 48 (2018): 138–149, 10.1016/j.ymben.2018.05.022. 29864583

[46] M. Hang , I. Gunsolus , H. Wayland , et al., “Impact of Nanoscale Lithium Nickel Manganese Cobalt Oxide (NMC) on the Bacterium Shewanella Oneidensis MR‐1,” Chemistry of Materials 28, no. 4 (2016): 1092–1100, 10.1021/acs.chemmater.5b04505.

[47] M. Li , D. Zhang , D. Holmes , et al., “Genetic and Transcriptomic Analysis of Microbial Electro‐Extraction for Releasing Metals From Spent Lithium‐Ion Batteries,” Bioresource Technology 440 (2026): 133479, 10.1016/j.biortech.2025.133479. 41083080

[48] A. Earl , M. Mohundro , I. Mian , and J. Battista , “The IrrE Protein of Deinococcus radiodurans R1 Is a Novel Regulator of recA Expression,” Journal of Bacteriology 184, no. 22 (2002): 6216–6224, 10.1128/jb.184.22.6216-6224.2002. 12399492 PMC151961

[49] H. Karlsson , S. Bucchianico , A. Collins , and M. Dusinska , “Can the Comet Assay be Used Reliably to Detect Nanoparticle‐Induced Genotoxicity?,” Environmental and Molecular Mutagenesis 56, no. 2 (2015): 82–96, 10.1002/em.21933. 25488706

[50] T. Chen , J. Wang , L. Zeng , et al., “Significant Rewiring of the Transcriptome and Proteome of an Escherichia coli Strain Harboring a Tailored Exogenous Global Regulator IrrE,” PLoS ONE 7, no. 7 (2012): 37126, 10.1371/journal.pone.0037126. PMC3390347 22792156

[51] M. Oparka , J. Walczak , D. Malinska , et al., “Quantifying ROS Levels Using CM‐H_2_ DCFDA and HyPer,” Methods 109 (2016): 3–11, 10.1016/j.ymeth.2016.06.008. 27302663

[52] T. Lin , W. Ding , L. Sun , L. Wang , C. Liu , and H. Song , “Engineered Shewanella Oneidensis‐Reduced Graphene Oxide Biohybrid With Enhanced Biosynthesis and Transport of Flavins Enabled a Highest Bioelectricity Output in Microbial Fuel Cells,” Nano Energy 50 (2018): 639–648, 10.1016/j.nanoen.2018.05.072.

[53] C. Lu , G. Bucher , and W. Sander , “Photoinduced Interactions Between Oxidized and Reduced Lipoic Acid and Riboflavin (Vitamin B_2_),” Chemphyschem 5, no. 1 (2004): 47–56, 10.1002/cphc.200300687. 14999843

[54] D. Coursolle , D. Baron , D. Bond , and J. Gralnick , “The Mtr Respiratory Pathway Is Essential for Reducing Flavins and Electrodes in Shewanella Oneidensis,” Journal of Bacteriology 192, no. 2 (2010): 467–474, 10.1128/JB.00925-09. 19897659 PMC2805334

[55] K. Kim , D. Raymond , R. Candeago , and X. Su , “Selective Cobalt and Nickel Electrodeposition for Lithium‐Ion Battery Recycling Through Integrated Electrolyte and Interface Control,” Nature Communications 12, no. 1 (2021): 6554, 10.1038/s41467-021-26814-7. PMC8590046 34772937

[56] Z. Liang , X. Ding , C. Cai , et al., “Acetate Acid and Glucose Assisted Subcritical Reaction for Metal Recovery From Spent Lithium Ion Batteries,” Journal of Cleaner Production 369 (2022): 133281, 10.1016/j.jclepro.2022.133281.

[57] V. Kumar , R. Mishra , G. Kaur , and D. Dutta , “Cobalt and Nickel Impair DNA Metabolism by the Oxidative Stress Independent Pathway,” Metallomics 9, no. 11 (2017): 1596–1609, 10.1039/c7mt00231a. 29058747

[58] H. Shi , X. Mao , F. Yang , et al., “Multi‐Scale Analysis of Acidophilic Microbial Consortium Biofilm's Tolerance of Lithium and Cobalt Ions in Bioleaching,” Journal of Hazardous Materials 474 (2024): 134764, 10.1016/j.jhazmat.2024.134764. 38824773

[59] C. Li , G. Dai , R. Liu , et al., “Separation and Recovery of Nickel Cobalt Manganese Lithium From Waste Ternary Lithium‐Ion Batteries,” Separation and Purification Technology 306 (2023): 122559, 10.1016/j.seppur.2022.122559.

[60] T. Liu , J. Chen , X. Shen , and H. Li , “Regulating and Regenerating the Valuable Metals From the Cathode Materials in Lithium‐Ion Batteries by Nickel‐Cobalt‐Manganese Co‐Extraction,” Separation and Purification Technology 259 (2021): 118088, 10.1016/j.seppur.2020.118088.

[61] W. Gao , J. Song , H. Cao , et al., “Selective Recovery of Valuable Metals From Spent Lithium‐Ion Batteries—Process Development and Kinetics Evaluation,” Journal of Cleaner Production 178 (2018): 833–845, 10.1016/j.jclepro.2018.01.040.

[62] X. Chen , B. Fan , L. Xu , T. Zhou , and J. Kong , “An Atom‐Economic Process for the Recovery of High Value‐Added Metals From Spent Lithium‐Ion Batteries,” Journal of Cleaner Production 112 (2016): 3562–3570, 10.1016/j.jclepro.2015.10.132.

[63] Y. Xin , X. Guo , S. Chen , J. Wang , F. Wu , and B. Xin , “Bioleaching of Valuable Metals Li, Co, Ni and Mn From Spent Electric Vehicle Li‐Ion Batteries for the Purpose of Recovery,” Journal of Cleaner Production 116 (2016): 249–258, 10.1016/j.jclepro.2016.01.001.

[64] M. Mandl , R. Lerchbammer , and E. Gerold , “Bioleaching of Lithium‐Ion Battery Black Mass: A Comparative Study on Gluconobacter Oxydans and Acidithiobacillus Thiooxidans,” Metals 15 (2025): 1112, 10.3390/met15101112.

[65] D. Ranganathan , J. Roy , B. Cao , and M. Srinivasan , “Facile Oxidative Recovery of Manganese as Electrochemically Active MnO_2_ From Spent Lithium‐Ion Battery Bioleachate,” ACS Sustainable Chemistry & Engineering 12, no. 28 (2024): 10475–10485, 10.1021/acssuschemeng.4c02595.

[66] N. Horeh , S. Mousavi , and S. Shojaosadati , “Bioleaching of Valuable Metals From Spent Lithium‐Ion Mobile Phone Batteries Using Aspergillus Niger,” Journal of Power Sources 320 (2016): 257–266, 10.1016/j.jpowsour.2016.04.104.

[67] N. Horeh and S. Mousavi , “Enhanced Recovery of Valuable Metals From Spent Lithium‐Ion Batteries Through Optimization of Organic Acids Produced by Aspergillus Niger,” Waste Management 60 (2017): 666–679, 10.1016/j.wasman.2016.10.034. 27825532

[68] Y. Yang , S. Lei , S. Song , W. Sun , L. Wang , and W. Manage , “Stepwise Recycling of Valuable Metals From Ni‐Rich Cathode Material of Spent Lithium‐Ion Battery,” Waste Management 102 (2020): 131–138, 10.1016/j.wasman.2019.09.044. 31677520

[69] F. Su , X. Zhou , X. Liu , et al., “Efficient Recovery of Valuable Metals From Spent Lithium‐Ion Batteries by Pyrite Method With Hydrometallurgy Process,” Chemical Engineering Journal 455 (2023): 140914, 10.1016/j.cej.2022.140914.

[70] J. Jung , P. Sui , and J. Zhang , “A Review of Recycling Spent Lithium‐Ion Battery Cathode Materials Using Hydrometallurgical Treatments,” Journal of Energy Storage 35 (2021): 102217, 10.1016/j.est.2020.102217.

