# Supplementary material for: Synthetic Shewanella‐Bacillus Microbial Consortia for Enhanced Metal Oxide Leaching From Spent Lithium‐Ion Batteries Using Brewing Wastewater as Leaching Agent
Source: Adv Sci (Weinh). 2026 Sep 13:e77699. Online ahead of print. doi: 10.1002/advs.77699 (PMC13572860; doi:10.1002/advs.77699)
Supplement: Supplementary file 1 [file ADVS-9999-e77699-s001.docx]

**Supporting Information (SI)**

**Synthetic *Shewanella-Bacillus*** **microbial consortia for enhanced metal ion leaching from spent lithium-ion batteries using brewing wastewater as leaching agent**

Baocai Zhang^1‡^, Chunxiao Qiao^1,2 ‡^, Huichun Liu^2^, Yiyun Wang^2^, Yan Liu^1^, Huan Yu^1^, Junqi Zhang^1^, Chao Li^1^, Deguang Wu^3^, Qulai Tang^3^, Zhiwen Wang^2, 4^, Danni Zhang^5^, Dake Xu^5^, Feng Li^2^, and Hao Song^1,2 *^

^1^ College of Life and Health Sciences, Northeastern University, Shenyang 110169, China.

^2^ State Key Laboratory of Synthetic Biology, and School of Synthetic Biology and Biomanufacturing, Tianjin University, Tianjin 300072, China.

^3^ Department of Brewing Engineering, Moutai Institute, Luban Avenue, Renhuai 564507, Guizhou, China.

^4^ Key Laboratory of Ministry of Education for Protection and Utilization of Special Biological Resources in Western China and School of Life Sciences, Ningxia University, Yinchuan 750021, China.

^5^ School of Materials Science and Engineering, Northeastern University, Shenyang 110819, China.

^‡^ Equal contribution.

* Corresponding author: H. S., E-mail: songhao@mail.neu.edu.cn

**Figure S1. The concentration of lithium, nickel, cobalt, and manganese leached from NCM that was catalyzed by the SO1-BS1microbial consortium.**

**Figure S2. The maximal rate of metal ions leaching, which was catalyzed by the microbial consortia of SO1-BS1, SO2-BS1, and SO2-BS2.**

**Figure S3.** **Representative fluorescence images of the comet assay of SO2 (in the absence of *irrE* expression), SO3 (in the presence of *irrE* expression), BS2 (in the absence of *irrE* expression) and BS3 (in the presence of *irrE* expression).**

**Figure S4. Cell growth profiles. a**, Cell growth profiles of strains SO2 (in the absence of Li^+^ and Mn^2+^), SO2 (in the presence of Li^+^ and Mn^2+^), and SO3 (in the presence of Li^+^ and Mn^2+^). **b**, Cell growth profiles of strains BS2 (in the absence of Li^+^ and Mn^2+^), BS2 (in the presence of Li^+^ and Mn^2+^), and BS3 (in the presence of Li^+^ and Mn^2+^).

**Figure S5. The concentration of Li, Ni, Co, Mn from** **brewing wastewater-reduced NCM catalyzed by SO3-BS3 microbial consortia**

**Figure S6. Schematic flowchart of the Stepwise Precipitation approach for the sequential recovery of high-purity Ni, Co, Mn, and Li from the leachate.** This work developed a Stepwise Precipitation approach to separate and recover high-purity metal salts from the leachate. 0.2 mol L^-1^ dimethylglyoxime solution was first added to the leachate at a stoichiometric molar ratio of 2:1 (dimethylglyoxime: Ni) to selectively precipitate Ni as Ni(C_4_H_6_N_2_O_2_)_2_. From the resulting filtrate, Co was subsequently precipitated as CoC_2_O_4_, by introducing 0.5 mol L^-1^ oxalic acid solution at a molar ratio of 1.2:1 (oxalic acid: Co). Both isolated precipitates were filtered. Subsequently, Mn was extracted from the solution using 70% saponified D2EHPA with 20 mmol L^-1^ HCl at an aqueous to organic phase ratio of 0.5. The Mn-loaded D2EHPA was stripped with 0.1 mol L^-1^ H_2_SO_4_ to obtain MnSO_4_. Finally, Li was recovered from the remaining aqueous phase as Li_3_PO_4_, by adding 0.5 mol L^-1^ H_3_PO_4_ solution at a molar ratio of 1.1:1 (H_3_PO_4_: Li).

**Table S1** Composition of the black mass used in this study.

| **Element** | **Content (w/w %)** |
| --- | --- |
| Li | 39.9 ± 3.0 |
| Ni | 12.6 ± 0.2 |
| Co | 4.7 ± 0.6 |
| Mn | 6.6 ± 0.2 |
| Fe | 1.5 ± 0.1 |
| Al | 0.4 ± 0.1 |
| Cu | 0.1 ± 0.1 |
| O* | 34.2 |

* The oxygen content was determined by difference from the total after subtracting metallic elements.

**Table S2.** Input cost and revenue summary of the proposed technology.

| **Process** | **Input/Output** | **Category** | **Quantity** | **Value** | **Cost/Revenue** | |
| --- | --- | --- | --- | --- | --- | --- |
| Seed culture process | Reagent cost | NaCl | 3.0 kg | 0.0592 $ kg^-1^ | 0.178 $ |  |
|  |  | Peptone | 3.0 kg | 7.11 $ kg^-1^ | 21.33 $ |  |
|  |  | Yeast extract | 1.5 kg | 4.44 $ kg^-1^ | 6.67 $ |  |
|  |  | Water | 0.65 t | 0.712 $ t^-1^ | 0.463 $ |  |
|  | Antibiotic cost | Chloramphenicol | 5.85 g | 118.52 $ kg^-1^ | 0.693 $ |  |
|  | Energy consumption | Culture | 19.5 kWh | 0.00438 $ kWh^-1^ | 0.0854 $ |  |
| Leaching process | Reagent cost | Black mass of NCM | 1 kg | 1.622 $ kg^-1^ | 1.622 $ |  |
|  | Antibiotic cost | Chloramphenicol | 25 g | 118.52 $ kg^-1^ | 2.96 $ |  |
|  | Inducer cost | IPTG | 0.596 kg | 800 $ kg^-1^ | 476.6 $ |  |
|  | Energy consumption | Heating | 8400 kWh | 0.00438 $ kWh^-1^ | 36.79 $ |  |
|  |  | Filtration | 3250 kWh | 0.00438 $ kWh^-1^ | 14.235 $ |  |
| Separation process | Reagent cost | H_3_PO_4_ | 6.25 kg | 1.33 $ kg^-1^ | 8.33 $ |  |
|  |  | Dimethylglyoxime | 0.168 kg | 2.203 $ kg^-1^ | 0.37 $ |  |
|  |  | Oxalic acid | 0.0476 kg | 0.481 $ kg^-1^ | 0.0229 $ |  |
|  |  | D2EHPA | 0.116 kg | 131.57 $ kg^-1^ | 15.262 $ |  |
|  |  | H_2_SO_4_ | 0.0705 kg | 0.382 $ kg^-1^ | 0.0269 $ |  |
|  |  | NaOH | 0.0232 kg | 0.504 $ kg^-1^ | 0.0117 $ |  |
|  |  | HCl | 0.0776 kg | 0.0185 $ kg^-1^ | 0.00144 $ |  |
|  | Energy consumption | Filtration | 3250 kWh | 0.00438 $ kWh^-1^ | 14.235 $ |  |
| Revenues | Recovered metal salts | Li_3_PO_4_ | 2355.44g | 0.2416 $ g^-1^ | 569.1 $ |  |
|  |  | Ni(C_4_H_6_N_2_O_2_)_2_ | 197.952 g | 0.0186 $ g^-1^ | 3.68 $ |  |
|  |  | CoC_2_O_4_ | 63.148 g | 0.0889 $ g^-1^ | 5.61 $ |  |
|  |  | MnSO_4_ | 104.655 g | 0.0127 $ g^-1^ | 1.33 $ |  |
|  | Treating brewing wastewater | Brewing wastewater | 5000 L | 5.18 $ t^-1^ | 25.92 $ |  |
| Profit | | |  |  | 5.754 $ |  |

**Table S3.** List of operating conditions and efficiencies for acid-based leaching of metals from NCM

| **Acid Type** | **Leaching agent** | **Temp.** | **Leaching efficiencies** | **Ref.** |
| --- | --- | --- | --- | --- |
| Inorganic acid | H_2_SO_4_ (2.5 M) + H_2_O_2_ (5%) | 50 ℃ | 97.2% Ni, 99.12% Co, 99.23% Mn, 99.54% Li | [1] |
|  | H_2_SO_4_ (3 M) + FeS_2_ (1:0.8 (g/g)) | 55 ℃ | 98.9% Ni, 99.5% Co, 98% Mn, 99.9% Li | [2] |
|  | H_2_SO_4_ (2 M) + H_2_O_2_ (0.97 M) | 80 ℃ | 100% Ni, Co, and Li and 94% Mn | [3] |
|  | HCl (3 M) | 70 ℃ | 100% Li, 99.7% Ni, 99.3% Co, 99.7% Mn | [4] |
| Organic acid | Malic acid (1.5 M) | 60 ℃ | 99.4% Li, <1.0% Ni, Co, and Mn | [5] |
|  | L-Malic acid (1.25 M) + H_2_O_2_ (1.5%) | 80 ℃ | 95.1% Ni, 95.3% Co, 96.2% Mn, 98.9% Li | [6] |
|  | Acetic acid (3.5 M) + H_2_O_2_ (4%) | 60 ℃ | 93.62% Co, 99.97% Li, 96.32% Mn, 92.67% Ni | [7] |
|  | Citric acid (1.5 M) + D-Glucose (0.5 g/g) | 80 ℃ | 91% Ni, 92% Co, 94% Mn, 99% Li | [8] |
|  | Ascorbic acid (0.4 M) + Acetic acid (2.5 M) | 80 ℃ | 95.27% Li, 97.23% Ni, 97.22% Co, 99% Mn | [9] |

**Table S4**. Bioleaching for recovery of metals from spent NCM

| **Microorganism** | **Leaching efficiencies** | **Ref.** |
| --- | --- | --- |
| *Acidithiobacillus thiooxidans* | ~10% Ni, ~10% Co, 20% Mn, 82% Li | [10] |
| *Acidithiobacillus thiooxidans* | 26% Ni, 14% Co, 37% Mn, 31% Li | [11] |
| *Acidithiobacillus ferrooxidans* | 25% Ni, 13% Co, 42% Mn, 57% Li | [12] |
| *Acidithiobacillus ferrooxidans* | 36.46% Ni, 37.29% Co, 33.38% Mn, 39.32% Li /cycle | [13] |
| *Acidithiobacillus ferrooxidans* | 27.87% Ni, 28.37% Co, 28.29% Mn, 33.73% Li /cycle | [14] |
| *Aspergillus niger* | 38% Ni, 45% Co, 70% Mn, 95% Li | [15] |
| *Aspergillus niger* | 54% Ni, 64% Co, 77% Mn, 100% Li | [16] |
| *Aspergillus niger* | 85% Ni, 87% Co, 90% Mn, 93% Li | [17] |

**Note S1**

The economic feasibility of the proposed technology was evaluated at an industrial scale, using 1 kg black mass of NCM as the functional unit for cost-benefit accounting. The techno-economic parameters utilized for this assessment are provided in **Table S2**. As a basis for these calculations, all reagent costs were estimated based on the purchase prices paid by our group to manufacturers in China and are reported in US dollars for reference (1 USD = 6.75 RMB; electricity: 0.00438 $ kWh^-1^; industrial water: 0.712 $ t^-1^). Energy consumption was calculated with reference to standard laboratory constant-temperature equipment (e.g., shakers and incubators), assuming a unit electricity demand of 0.005 kWh h^-1^ L^-1^. A stepwise precipitation approach was adopted for the downstream separation of metal products (**Figure S6**). The revenue from wastewater treatment was credited at 5.18 $ per ton of brewing wastewater according to the treatment fee charged by the local brewery to the wastewater treatment plant.

Based on these fundamental parameters, the economic assessment was systematically structured around comprehensive cost inputs and revenues. On the cost side, total operational expenses were categorized into three sequential stages, the seed culture, leaching, and separation processes, which comprehensively covered basal reagents (e.g., culture media components, the NCM black mass, and chemical precipitants/extractants), reagents for plasmid maintenance and gene expression (e.g., chloramphenicol and IPTG), and process-specific energy consumption (cultivation, heating, and filtration). On the revenues side, the gross revenue was derived from two distinct streams, comprising the commercial value of the recovered high-purity metal salts (Li_3_PO_4_, Ni(C_4_H_6_N_2_O_2_)_2_, CoC_2_O_4_, and MnSO_4_) and the environmental revenue from treating brewing wastewater. Therefore, the net profit was calculated by balancing these aggregated multi-stage costs against the dual-stream revenues, expressed by the formula:

Profit (1 kg black mass of NCM) = R _Recovered metal salts_ + R _Treating brewing wastewater_ - C _Seed culture process_ - C _Leaching process_ - C _Separation process_

**Supplementary References**

[1]	C. Li, G. Dai, R. Liu, C. Wang, S. Wang, Y. Ju, H. Jiang, S. Jiao, C. Duan, *Sep. Purif. Technol.* **2023**, *306*, 122559, <https://doi.org/https://doi.org/10.1016/j.seppur.2022.122559>.

[2]	F. Su, X. Zhou, X. Liu, J. Yang, J. Tang, W. Yang, Z. Li, H. Wang, Y. Ma, *Chem. Eng. J.* **2023**, *455*, 140914, <https://doi.org/https://doi.org/10.1016/j.cej.2022.140914>.

[3]	Y. Yang, S. Lei, S. Song, W. Sun, L. Wang, *Waste Manage.* **2020**, *102*, 131, <https://doi.org/https://doi.org/10.1016/j.wasman.2019.09.044>.

[4]	T. Liu, J. Chen, X. Shen, H. Li, *Sep. Purif. Technol.* **2021**, *259*, 118088, <https://doi.org/https://doi.org/10.1016/j.seppur.2020.118088>.

[5]	H. Yu, D. Wang, S. Rao, L. Duan, C. Shao, X. Tu, Z. Ma, H. Cao, Z. Liu, *Int. J. Miner., Metall. Mater.* **2024**, *31* (4), 688, https://doi.org/10.1007/s12613-023-2741-3.

[6]	E. Fan, J. Yang, Y. Huang, J. Lin, F. Arshad, F. Wu, L. Li, R. Chen, *ACS Appl. Energy Mater.* **2020**, *3* (9), 8532, <https://doi.org/10.1021/acsaem.0c01166>.

[7]	W. Gao, J. Song, H. Cao, X. Lin, X. Zhang, X. Zheng, Y. Zhang, Z. Sun, *J. Cleaner Prod.* **2018**, *178*, 833, <https://doi.org/https://doi.org/10.1016/j.jclepro.2018.01.040>.

[8]	X. Chen, B. Fan, L. Xu, T. Zhou, J. Kong, *J. Cleaner Prod.* **2016**, *112*, 3562, <https://doi.org/10.1016/j.jclepro.2015.10.132>.

[9]	J. Zhang, X. Hu, T. He, X. Yuan, X. Li, H. Shi, L. Yang, P. Shao, C. Wang, X. Luo, *Waste Manage.* **2023**, *165*, 19, https://doi.org/https://doi.org/10.1016/j.wasman.2023.04.020.

[10]	Y. Xin, X. Guo, S. Chen, J. Wang, F. Wu, B. Xin, *J. Cleaner Prod.* **2016**, *116*, 249, <https://doi.org/10.1016/j.jclepro.2016.01.001>.

[11]	M. Mandl, R. Lerchbammer, E. Gerold, *Metals* **2025**, *15* (10), 1112, <https://doi.org/10.3390/met15101112>.

[12]	M. Nazerian, N. Horeh, S. Mousavi, *Korean J. Chem. Eng.* **2023**, *40* (3), 584, <https://doi.org/10.1007/s11814-022-1257-2>.

[13]	J. Roy, M. Srinivasan, B. Cao, *ACS Sustainable Chem. Eng.* **2021**, *9* (8), 3060, <https://doi.org/10.1021/acssuschemeng.0c06573>.

[14]	D. Ranganathan, J. Roy, B. Cao, M. Srinivasan, *ACS Sustainable Chem. Eng.* **2024**, *12* (28), 10475, <https://doi.org/10.1021/acssuschemeng.4c02595>.

[15]	N. Horeh, S. Mousavi, S. Shojaosadati, *J. Power Sources* **2016**, *320*, 257, <https://doi.org/10.1016/j.jpowsour.2016.04.104>.

[16]	N. Horeh, S. Mousavi, *Waste Manage.* **2017**, *60*, 666, <https://doi.org/10.1016/j.wasman.2016.10.034>.

[17]	N. Horeh, S. Mousavi, M. Baniasadi, *J. Cleaner Prod.* **2018**, *197*, 1546, <https://doi.org/10.1016/j.jclepro.2018.06.299>.
